# Protein disulfide isomerase blocks the interaction of LC3II-PHB2 and promotes mTOR signaling to regulate autophagy and radio/chemo-sensitivity

**DOI:** 10.1038/s41419-022-05302-w

**Published:** 2022-10-06

**Authors:** Ruru Wang, Yajing Shang, Bin Chen, Feng Xu, Jie Zhang, Zhaoyang Zhang, Xipeng Zhao, Xiangbo Wan, An Xu, Lijun Wu, Guoping Zhao

**Affiliations:** 1 High Magnetic Field Laboratory, Key Laboratory of High Magnetic Field and Ion Beam Physical Biology, Chinese Academy of Sciences, Anhui Province Key Laboratory of Environmental Toxicology and Pollution Control Technology, Hefei Institutes of Physical Science, Chinese Academy of Sciences, Hefei, Anhui 230031 China; 2 University of Science and Technology of China, Hefei, Anhui 230026 China; 3 Anhui Medical University, Hefei, Anhui 230032 China; 4 Information Materials and Intelligent Sensing Laboratory of Anhui Province, Institutes of Physical Science and Information Technology, Anhui University, Hefei, Anhui 230601 China; 5 The Sixth Affiliated Hospital, Sun Yat-sen University, Guangzhou, Guangdong 510275 China

**Keywords:** Radiotherapy, Tumour-suppressor proteins

## Abstract

Protein disulfide isomerase (PDI) is an endoplasmic reticulum (ER) enzyme that mediates the formation of disulfide bonds, and is also a therapeutic target for cancer treatment. Our previous studies found that PDI mediates apoptotic signaling by inducing mitochondrial dysfunction. Considering that mitochondrial dysfunction is a major contributor to autophagy, how PDI regulates autophagy remains unclear. Here, we provide evidence that high expression of PDI in colorectal cancer tumors significantly increases the risk of metastasis and poor prognosis of cancer patients. PDI inhibits radio/chemo-induced cell death by regulating autophagy signaling. Mechanistically, the combination of PDI and GRP78 was enhanced after ER stress, which inhibits the degradation of AKT by GRP78, and eventually activates the mTOR pathway to inhibit autophagy initiation. In parallel, PDI can directly interact with the mitophagy receptor PHB2 in mitochondrial, then competitively blocks the binding of LC3II and PHB2 and inhibits the mitophagy signaling. Collectively, our results identify that PDI can reduce radio/chemo-sensitivity by regulating autophagy, which could be served as a potential target for radio/chemo-therapy.

## Introduction

Colorectal cancer (CRC) has been identified as the third largest cancer in the world, and the global incidence rate and mortality are still rising [1]. Currently, chemo-therapy or radio-therapy combined with surgery is used as the backbone for treating metastatic CRC, which, unfortunately, shows limited efficacy [2, 3]. Autophagy, as type II programmed cell death, is a complex process of intracellular degradation of senescent or malfunctioning organelles. Recently, autophagy inhibitors or inducers have overcome multidrug or radio-resistance of cancer cells, suggesting that autophagy is a new target for cancer therapy [4–6]. Up to now, the dual role of autophagy both in cancer progression and inhibition remains controversial.

Protein disulfide isomerase (PDI), also known as prolyl 4-hydroxylase beta, is a highly abundant multifunctional enzyme that belongs to the PDI family, which is classically characterized by endoplasmic reticulum (ER) localization and redox activity [7–9]. PDI family proteins, such as AGR2, PDI, PDIA3, PDIA4, and PDIA6 are reportedly upregulated in cancers [10–16], which are correlated with cancer progression and metastatic disease in pancreatic [17], kidney renal clear cell carcinoma (KIRC) [11], ovarian cancers [18], glioblastoma [19–21] and lung adenocarcinoma [22]. Increasing evidence suggests that PDI supports the survival and progression of several cancers by regulating endoplasmic reticulum stress (ERS), apoptosis, DNA repair, and autophagy [7]. For example, PDI knockout reduces DNA repair after ionizing radiation (IR) and enhances the killing ability of radiation on GBM cells [23]. Besides, knockdown of PDI can remove survival responses to ERS, thereby promoting melanoma cell death [24]. Inhibition of PDI results in increased autophagy, and prevents the death of dopaminergic neurons [25]. PDI inhibitors have been shown to enhance chemo-sensitivity both in vitro and in vivo [26, 27]. Given the emerging role of PDI in cancer, PDI is generally described as a proto-oncogene and potential cancer biomarker [20, 28]. However, the expression of PDI is also correlated with positive clinical outcomes. For example, breast patients with elevated levels of autoantibodies against PDI were found to have higher survival [29]. PDI mediates the decrease of H_2_O_2_ level in the ER of cervical cancer cells and inhibits cell migration, invasion, and tumor growth [30]. In addition, overexpression of PDI family protein ERp29 significantly inhibits proliferation and tumorigenesis by regulating ERS signals and mesenchymal-epithelial transition in breast cancer cells [31].

One explanation for the disparate prognostic correlation between PDI family proteins and patient outcomes may involve the differences in PDI cellular localization, and depends on whether the cellular concentration of the PDIs can reach a certain threshold to initiate tumor suppressor function. For example, PDI prevents apoptotic signaling associated with ERS and protein misfolding in various in vivo and in vitro models, however, higher concentrations of purified PDI (0.5–1.5 μM) promote MOMP in vitro [32]. Tiemann et al. found that AGR2 in the extracellular promoted the phosphorylation of RICTOR and inhibited tumor metastasis, whereas intracellular AGR2 antagonized its level and phosphorylation and promoted tumor metastasis. This indicates that the PDI family protein can participate in the regulation of tumor progression through different cell localizations or intracellular concentrations [33]. Another possibility is that PDI plays a double-sided role in autophagy signaling [4, 34]. Studies have reported that PDI may participate in autophagy regulation through GRP78 or ERS to regulate tumor development [7, 35–37]. However, the detailed molecular mechanisms of PDI-mediated autophagy are not completely known.

In this study, we set out to explore the regulatory mechanism of PDI in autophagy and the role of PDI-mediated autophagy in radio/chemo-therapy sensitivity of CRC. Our study demonstrated that PDI regulates autophagy through the AKT-mTOR pathway and binds with PHB2 to inhibit the autophagy pathway. We further extended the mechanistic exploration and found that PDI could inhibit the death of CRC cells by inhibiting the autophagy level, which reduced the sensitivity of radio/chemo-therapy.

## Results

### PDI is elevated in human colorectal cancer tissues and is associated with a poor prognosis

**Fig. 1:**
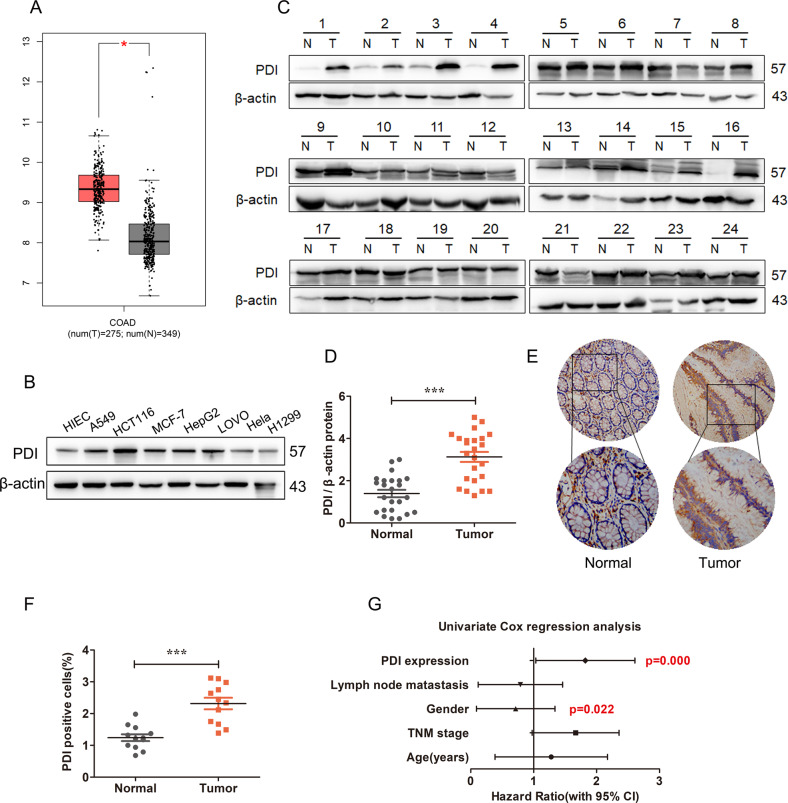
PDI is elevated in human colorectal cancer tissues and is associated with poor prognosis. **A** TCGA database was used to analyze the expression of PDI mRNA in CRC tissue (T) and normal tissue (N). **B** Expression of PDI protein in normal intestinal epithelial and tumor cells. **C**, **D** The level of PDI protein in 30 pairs of colorectal cancer tissues (T) and paired adjacent normal tissues (N) was analyzed by western blot (**C**). Quantification of protein levels (**D**) was normalized to those of β-actin. **E**, **F** Represent immunohistochemically staining (**E**) and IHC score (**F**) of PDI expression in CRC and adjacent normal tissues. Original magnification, ×40 (outside) or ×400 (inset). Scale bar = 50 μm. **G** Forest plots for univariate analysis of the correlation between clinicopathological features of colon cancer and mortality. Data are presented as the mean ± SD (***P < 0.001 by Student’s tests).

Our previous study identified genes that were differentially expressed between CRC cells and IR treatment using RNA sequencing analyses [38]. PDI was one of the upregulated genes identified in CRC cells (Supplementary Fig. S1a). To investigate the role of PDI in CRC, we analyzed the transcriptional datasets assembled from clinical samples in the public database GEPIA (http://gepia.cancer-pku.cn) and discovered that the transcriptional levels of PDI were dramatically increased in various cancers, especially in CRC cancer tissues (Fig. 1A and Supplementary Fig. S1b–d). Further analysis showed that the level of PDI protein in CRC tissues (*n* = 30) and five tumor cell lines was also higher than that in paired adjacent normal tissues and normal human intestinal epithelial cells (HIEC), respectively (Fig. 1B–D). Consistent with these results, immunohistochemistry staining of CRC showed strongly positive PDI staining in clinical CRC tissues, but weakly positive or negative PDI staining in adjacent normal tissues (Fig. 1E, F). To clarify the clinical relevance of PDI, we tested the potential association between PDI expression and clinic pathological features in 60 CRC patients. As shown in Table 1, the expression of PDI is significantly correlated with the survival status of patients, but not with gender, age, lymph node metastasis, or age. Importantly, univariate cox regression analysis showed that both PDI expression and gender were important predictors of overall survival in patients with CRC (Fig. 1G).

**Table 1:** Demographic and clinicopathological parameters of patients with hepatocellular carcinoma (data from 30 clinical patients).

Characteristic	PDI expression		*χ*^2^	*P* value
	High (*n* = 17)	Low (*n* = 13)		
Age (years)				
<60	9	6		
≥60	8	7	0.24	0.624
TNM stage				
I	4	5		
II	3	4		
III	4	3		
IV	6	1	3.497	0.321
Gender				
Male	11	5		
Female	6	8	2.039	0.153
Lymph node metastasis				
Positive	3	0		
Negative	14	13	2.549	0.11
Survival status				
Death	5	0		
Survival	12	13	19.714	0.032

### PDI decreases radio/chemo-sensitivity in cancer cells

**Fig. 2:**
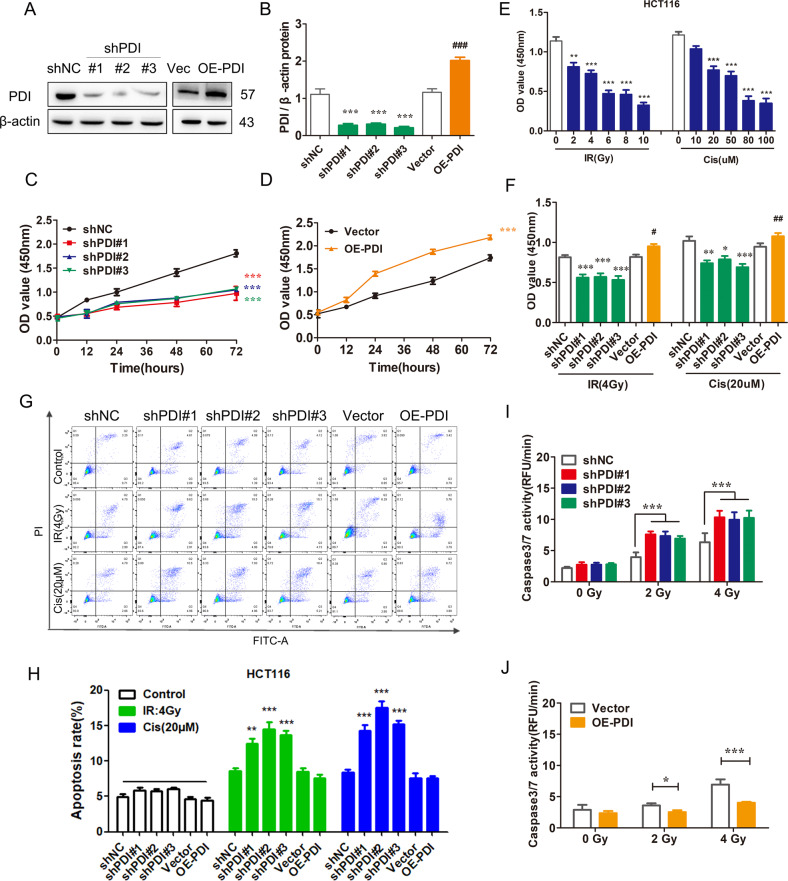
PDI decreases radio/chemo-sensitivity in cancer cells. **A**, **B** Immunoblot analysis of PDI protein in PDI knockdown or overexpression HCT116 (**A**), and semi-quantitative statistics using image software (**B**). **C**, **D** CCK-8 assay analysis of the impact of knockdown (**C**) or overexpression of PDI (**D**) on the viability of HCT116 cells. **E**, **F** CCK-8 assay analysis of HCT116 cells after γ-ray or cisplatin for 24 h. **G**, **H** The apoptotic cells were assessed by annexin V-FITC/PI staining after being treated with γ-ray (4 Gy) irradiation for 24 h (**G**), flowjo software performs data statistics. **I**, **J** Caspase3/7 kit was used to detect the effect of knockdown or overexpression of PDI combined with γ-ray (4 Gy) or cisplatin (20 μM) for 24 h on the apoptosis of HCT116 cells. Data of at least three independent experiments performed in duplicate are presented as mean ± SEM. shNC shRNA control, shPDI#1/2/3 shRNA-1/2/3 targeting PDI, Vector control plasmid, OE-PDI PDI overexpression plasmid. *P < 0.01, **P < 0.01, ***P < 0.001 compared with shNC. ^##^*P* < 0.01, ^###^*P* < 0.001 compared with control vector plasmid transfected cells.

To further investigate the role of PDI in CRC, PDI knockdown or overexpressed cell lines were established in HCT116, MEF, and A549 cells (Fig. 2A, B and Supplementary Fig. S2a, b). MEF cells used here were immortalized by infection with SV40 large T antigen-expressing lentivirus, which has the characteristics of cancer cells [39]. Then, we used CCK-8 analysis to evaluate the effect of PDI on the viability of cells. The results showed that cell viability was decreased in cells following PDI knockdown, but increased in cells overexpressing PDI compared to the corresponding control groups (Fig. 2C, D and Supplementary Fig. S2c, d). Next, to further validate the oncogenic activity of PDI in radio/chemo-sensitivity, the cells were treated with radio/chemo-therapy. The results indicated that knockdown of PDI significantly increased radiation (IR) or cisplatin (Cis) induced cytotoxic effects in HCT116 cell viability, whereas PDI overexpression showed the opposite effect (Fig. 2E, F). Consistently, flow cytometry analysis with annexin V/PI double staining showed that PDI knockdown increased the apoptotic rate of HCT116 and A549 cells compared to control shRNA after IR/Cis treatment (Fig. 2G, H and Supplementary Fig. S2e). Caspase activity analysis also showed an increased apoptotic rate in PDI knockdown cells, and a decreased apoptotic rate in PDI overexpressing cells after IR treatment (Fig. 2I, J and Supplementary Fig. S2f–h). Similar results were obtained in a colony formation assay in HCT116 cells (Supplementary Fig. S2i, j). These data indicate that PDI plays a role in promoting cell proliferation as an oncogene, and PDI inhibits IR/Cis-induced apoptotic cell death.

### PDI regulates radio/chemo-sensitivity by regulating autophagy

**Fig. 3:**
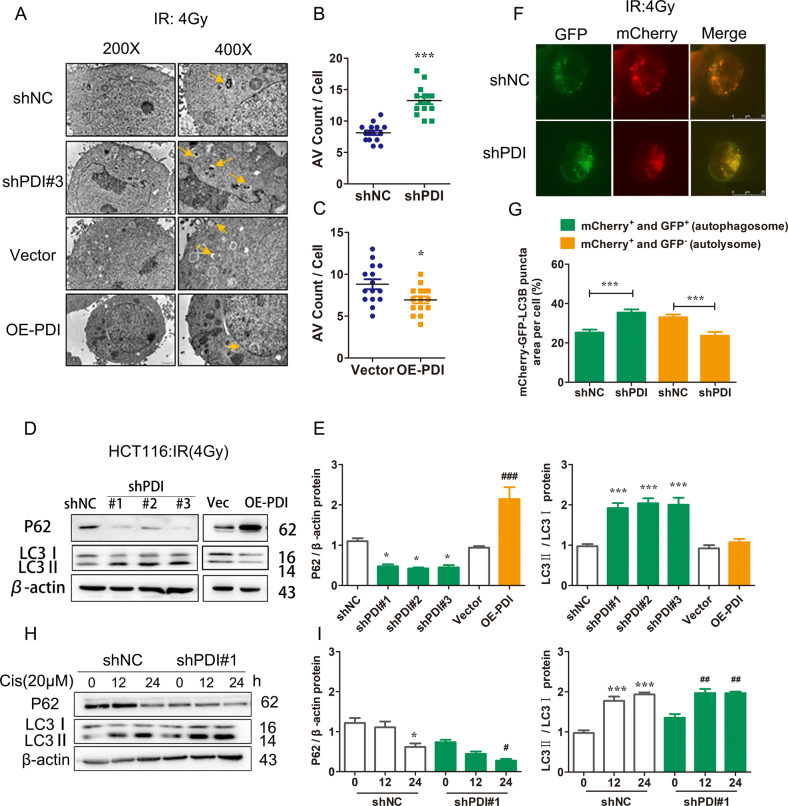
PDI regulates radio/chemo-sensitivity by regulating autophagy. **A**–**C** HCT116 cells were treated with γ-ray (4 Gy) irradiation for 24 h and were then subjected to transmission electron microscopy assay. Scale bar = 2 μm (left) or scale bar = 1 μm (right). Arrows indicate autophagosomes or autolysosomes (**A**). The average number of autophagosomes (**B**) or autolysosomes (**C**) per cell was quantified. **D**, **E** Western blot showed levels of autophagy-related proteins in PDI knockdown or overexpression cells after being treated with IR for 24 h and the results were quantified. **F**, **G** HCT116 cells or PDI knockdown cells transiently transfected with mCherry-GFP-LC3B plasmid and after 24 h were treated with γ-ray (4 Gy) irradiation for 24 h. The distribution of LC3II was visualized by confocal microscopy (up) and the average number of GFP-LC3 dots per cell was quantified (down). Scale bar = 25 μm. **H**, **I** Western blot showed levels of autophagy-related proteins in HCT116 cells after being treated with cisplatin (20 μM) for 24 h. Data of at least three independent experiments performed in duplicate are presented as mean ± SEM. *P < 0.01, **P < 0.01, ***P < 0.001 compared with shNC. ^#^*P* < 0.05, ^##^*P* < 0.01, ^###^*P* < 0.001 compared with control vector plasmid transfected cells.

Next, we detected whether autophagy signaling was involved in PDI-mediated radio/chemo-sensitivity. TEM assays and western blot is presented to show that PDI knockdown combined with IR treatment increased the formation of autophagosomes (Fig. 3A–C), enhance light chain 3II (LC3II)/I ratio, and decrease p62 protein level compared with control cells upon radiation in HCT116 cells (Fig. 3D, E and Supplementary Fig. S3a). Increased LC3II/I ratio and decreased p62 protein level were also found in A549 PDI knockdown cells. Moreover, the number of green fluorescent punctas was strongly increased under IR treatment in PDI knockdown HCT116 cells (Fig. 3F, G). Similarly, compared with the control group, knockdown of PDI can also enhance cisplatin-induced autophagy (Fig. 3H, I). However, overexpression of PDI has less effect on radio/chemo-induced autophagy. Those results demonstrate that knockdown PDI can accelerate the process of autophagy during cell treatment with radio/chemo-therapy.

### Knockdown PDI promotes chemo/radio-therapy sensitivity

**Fig. 4:**
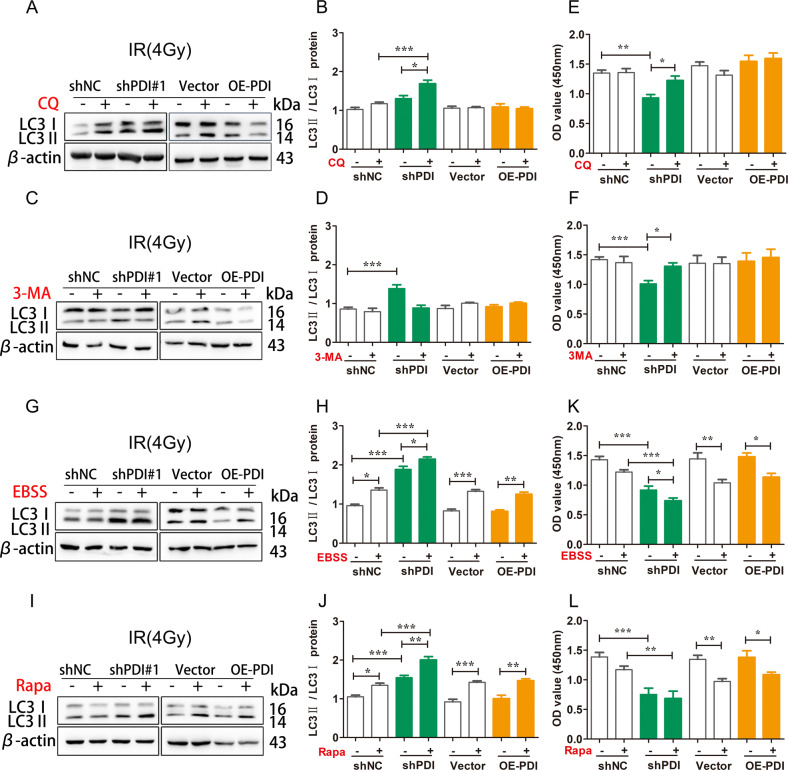
Knockdown PDI to promote chemo/radio-therapy sensitivity. **A**–**D** HCT116 cells were pretreated with or without 10 µM CQ (**A**) or 5 mM 3-MA (**C**) for 2 h, and then radiated with 4 Gy γ-ray for 24 h. LC3II accumulation was measured using WB. **E**, **F** CCK-8 assay analysis of the impact of cell viability pretreated with or without CQ (**E**) or 3-MA (**F**) for 2 h. **G**–**J** HCT116 cells were irradiated with 4 Gy γ-ray for 24 h, after that treated with 10 µm EBSS (**G**) or rapamycin (**H**) that induced autophagy. LC3II accumulation was measured using WB. K-L CCK-8 assay analysis of the impact of cell viability treated with EBSS (**K**) or rapamycin (**L**). Data of three independent experiments are presented as mean ± SEM. *P < 0.05, **P < 0.01, ***P < 0.01 compared with control.

To further investigate the mechanism of PDI participating in autophagy, the autophagy inhibitor chloroquine (CQ) and 3-methyladenine (3-MA) prevent the end-stage or early-stage process of autophagy was used. As shown in Fig. 4A–D and Supplementary Fig. S4a, b, the combination of CQ pretreatment and IR/Cis significantly increased the accumulation of LC3II in PDI knockdown cells, but not in overexpression cells, compared with the IR/Cis alone. Knockdown or overexpressing PDI did not affect autophagy after inhibiting early autophagy by 3-MA pretreatment (Fig. 4E, F and Supplementary Fig. S4c, d). Importantly, both 3-MA and CQ decreased radio/chemo-resistance in PDI knockdown cells but not overexpression cells (Fig. 4G–J and Supplementary Fig. S4e, f). Moreover, the autophagy inducer rapamycin or EBSS combined with IR/Cis treatment can significantly enhance the autophagy level and decrease cell viability in cells (Fig. 4K, L and Supplementary Fig. S4g, h). These results signified that overexpression of PDI increases cell viability by attenuating autophagy signaling, and thus enhances cellular radio/chemo-therapy resistance, and that the function of PDI in radio/chemo-resistance is mainly autophagy-dependent.

As PDI plays an important role in cells depending on its enzyme activity, we also studied whether the enzyme activity of PDI was involved in autophagy signaling. PDI enzyme activity inhibitors bacitracin and securinine were used. We found that bacitracin and securinine failed to affect the autophagy level and cell viability of cells upon radiation. (Supplementary Fig. S4i–l), indicating PDI-induced autophagy was independent of its catalytic activity.

### PDI interacts with GRP78, leads to ERS, and activates AKT/mTOR signaling pathway

**Fig. 5:**
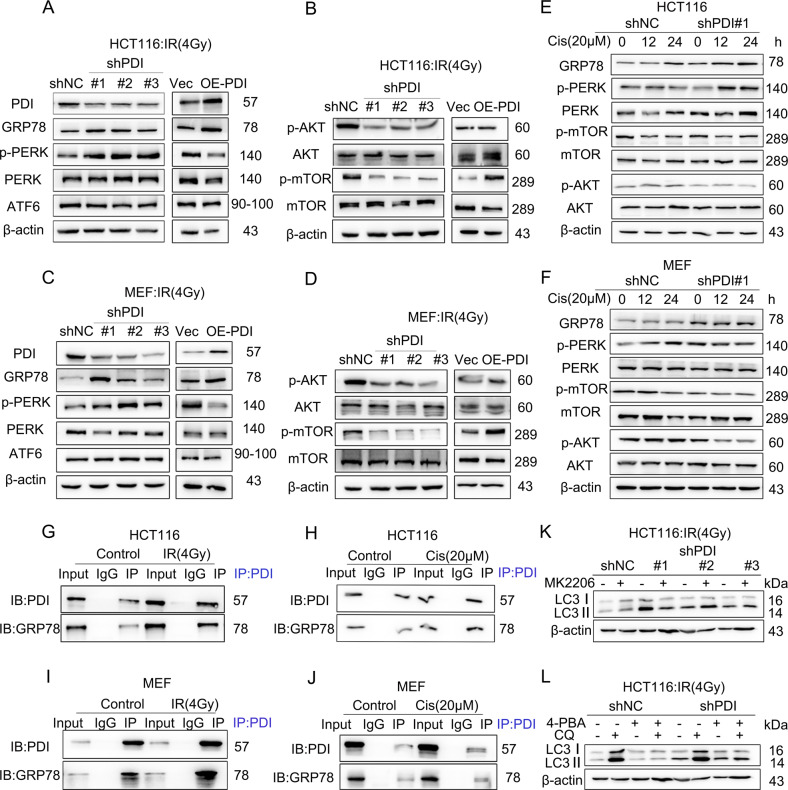
PDI interacts with GRP78, leads to ERS, and activates AKT/mTOR signaling pathway. **A**–**D** Immunoblot analysis showing the levels of ERS pathway-related proteins GRP78, PERK, ATF6 (**A**, **C**) or AKT/mTOR pathway-related proteins (**B**, **D**) in PDI knockdown or overexpression HCT116 or MEF cells treated with γ-ray (4 Gy) or cisplatin (20 μM) irradiation for 24 h. **E**, **F** Immunoblot analysis showing the levels of ERS pathway-related proteins and AKT/mTOR pathway-related proteins in PDI knockdown or overexpression MEF cells treated with γ-ray irradiation (**E**) or cisplatin (**F**) for 24 h. **G**, **H** The existence of PDI in the co-precipitated complexes was confirmed by western blotting, IgG was employed as the negative control. Western blotting shows the association of PDI with GRP78 after Co-IP in HCT116 cells were treated with γ-ray (4 Gy) irradiation (**G**) or cisplatin (20 μM) (**H**) for 24 h. **I**, **J** The existence of PDI in the co-precipitated complexes was confirmed by western blotting, IgG was employed as the negative control. Western blotting shows the association of PDI with GRP78 after Co-IP in MEF cells were treated with γ-ray (4 Gy) irradiation (**I**) or cisplatin (20 μM) (**J**) for 24 h. **K**, **L** HCT116 cells were pretreated with or without 5 mM MK2206 (**K**) or 10 µM 4-PBA (**L**) for 2 h, then irradiated with 4 Gy γ-ray for 24 h. LC3II accumulation was measured using western blotting.

To investigate the mechanism of PDI participating in autophagy, we conducted an RNA sequencing analysis to identify potential pathways involved in PDI-mediated autophagy signaling. The results indicated that AKT/mTOR pathway mainly participates in PDI-mediated autophagy after radiation treatment (Supplementary Fig. S5a). As shown in Fig. 5A–D, increased levels of GRP78, a well-characterized marker for ERS [40], were observed after IR/Cis treatment in cells. The level of phosphorylated PERK, but not the ER membrane-spanning UPR receptor protein ATF6 was increased upon IR/Cis treatment. PDI knockdown promoted IR/Cis-induced ERS and decreased the levels of phosphorylated AKT and mTOR but not their total protein levels, whereas PDI overexpression promoted AKT and mTOR phosphorylation after IR/Cis in HCT, A549, and MEF cells (Fig. 5E, F and Supplementary Fig. S5b). Knockdown of PDI also increased the protein expression level of ULK1 after IR/Cis treatment (Supplementary Fig. S5c). These results indicated that PDI-mediated autophagy induced by IR/Cis in an AKT-mTOR-ULK1 signaling-dependent manner. To further explore the mechanism, we performed an online data analysis (https://string-db.org/) and observed that GRP78 may function as a PDI substrate protein (Supplementary Fig. S5d). We confirmed the interaction between endogenous PDI and GRP78 using a co-immunoprecipitation (Co-IP) assay, and the interaction of PDI and GRP78 was notably promoted by IR/Cis stimulation (Fig. 5G–J). Similar results were also observed in the immunofluorescence assay, where more endogenous GRP78 colocalized with PDI in the cytoplasm upon IR/Cis stimulation (Supplementary Fig. S5e, f).

Next, we treated cells with the AKT-specific inhibitor MK2206 or ERS inhibitor 4-PBA, and found that MK2206 and 4-PBA treatment did not completely suppress PDI knockdown-mediated autophagy (Fig. 5K, L), indicating that except for the classical mTOR pathway, other signaling pathways may be involved in PDI-mediated autophagy.

### PDI prevents autophagy by regulating mitophagy

**Fig. 6:**
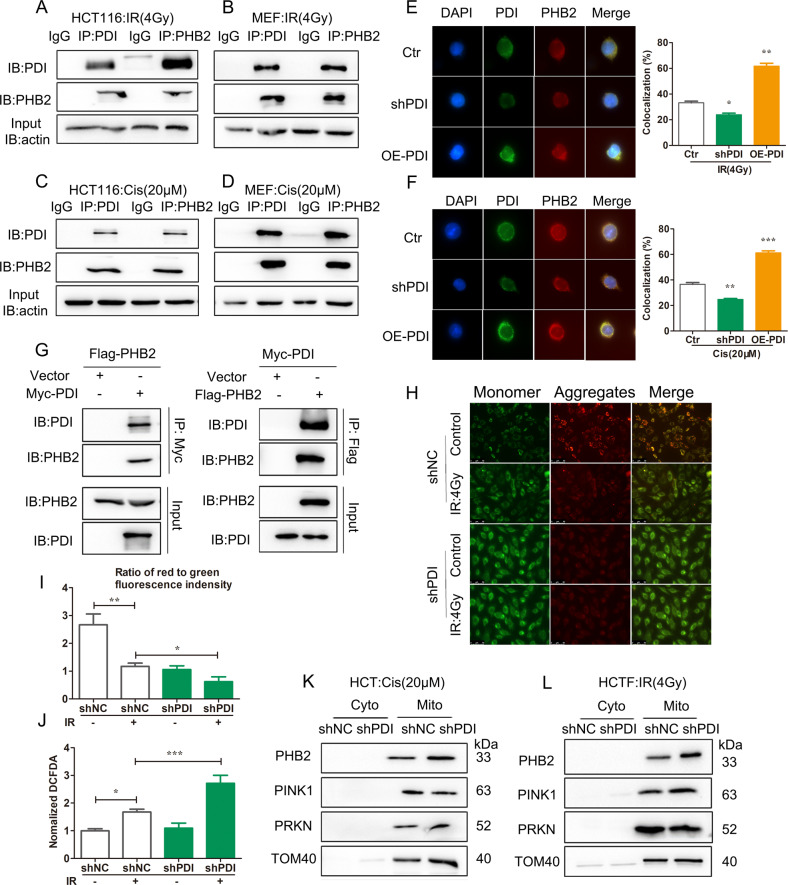
PDI prevents autophagy by regulating mitophagy. **A**–**D** The interaction between PDI and PHB2 was detected by immunoprecipitation after the treatment with γ-ray (4 Gy) irradiation (**A**, **B**) or 20 μM cisplatin (**C**, **D**) for 24 h in HCT116 and MEF cells. **E**, **F** Immunofluorescence microscope analysis of co-localization of PDI (red) and PHB2 (green) in PDI knockdown or PDI overexpression HCT116 cells after 24 h of treatment with IR (4 Gy) (**E**) or cisplatin (20 μM) (**F**). Blue DAPI staining was used to stain the cell nucleus. Scale bar = 25 μm. **G** The interaction between PDI and PHB2 was detected by Flag-pull down in vitro. Purified Flag-PHB2 or Myc-PDI immobilized on the beads was incubated with purified Myc-PDI or Flag-PHB2. Input and bead-bound proteins were analyzed by immunoblotting with anti-PDI or anti-PHB2 antibodies. **H** The changes in the MMP of HCT116 cells before and after γ-ray (4 Gy) irradiation for 24 h were detected by the JC-1 probe. **I** Statistical map of MMP. **J** The expression of ROS in HCT116 cells after γ-ray (4 Gy) irradiation for 24 h. **K**, **L** Expression of mitophagy-related proteins in mitochondria treated with γ-ray (4 Gy) irradiation (**K**) after 24 h in HCT116 cells or treated with cisplatin (20 μM) after 24 h in MEF cells (**L**). Data were pooled from three independent experiments and the results are represented as mean ± SD; *P < 0.05, **P < 0.01, ***P < 0.001.

To determine whether PDI is associated with other factors related to post-radiation autophagy, we conducted Co-IP with the PDI antibody and then performed liquid chromatography with tandem mass spectrometry (LC-MS/MS). The LC-MS/MS data identified a set of proteins that are commonly involved in the autophagy pathway based on GO analysis. Among those candidate proteins, PHB2, which is a mitophagy receptor strongly associated with mitochondrial dysfunction, particularly with autophagy processes, got the highest score (Supplementary Fig. S6a). Database analysis also presented a positive correlation between PDI and PHB2, compared with that of LC3 (Supplementary Fig. S6b, c). As shown in Fig. 6A–D, PHB2 stably interacted with PDI as validated by Co-IP in cells after radio/chemo-treatment. Additionally, immunofluorescence assay showed that PDI (green) and PHB2 (red) were colocalized upon IR/Cis treatment in HCT116 cells. (Fig. 6E, F). To confirm this result, we performed an in vitro binding assay with purified Myc-PDI and Flag-PHB2 proteins, and the results revealed that PDI is directly associated with PHB2 (Fig. 6G).

Previous studies have shown that mitochondrial depolarization is a prerequisite for PHB2 expression on the outer mitochondrial membrane (MOM). Therefore, we sought to determine whether PDI mediated mitochondrial depolarization. Our experiments confirmed that PDI knockdown increased radiation-induced oxidative stress and decreased MOM potential, resulting in cellular mitochondrial depolarization (Fig. 6H–J). It was also found that although the total protein levels of PHB2 were not changed (Supplementary Fig. S6d, e), the expression level of PHB2 but not PINK1/Parkin in purified mitochondria was increased after IR or Cis treatment in PDI knockdown cells compared with control vector cells (Fig. 6K, L). This indicated that the binding degree of PDI and PHB2 is the key factor to regulate autophagy.

### PDI interacts with PHB2 to attenuate autophagic activity

**Fig. 7:**
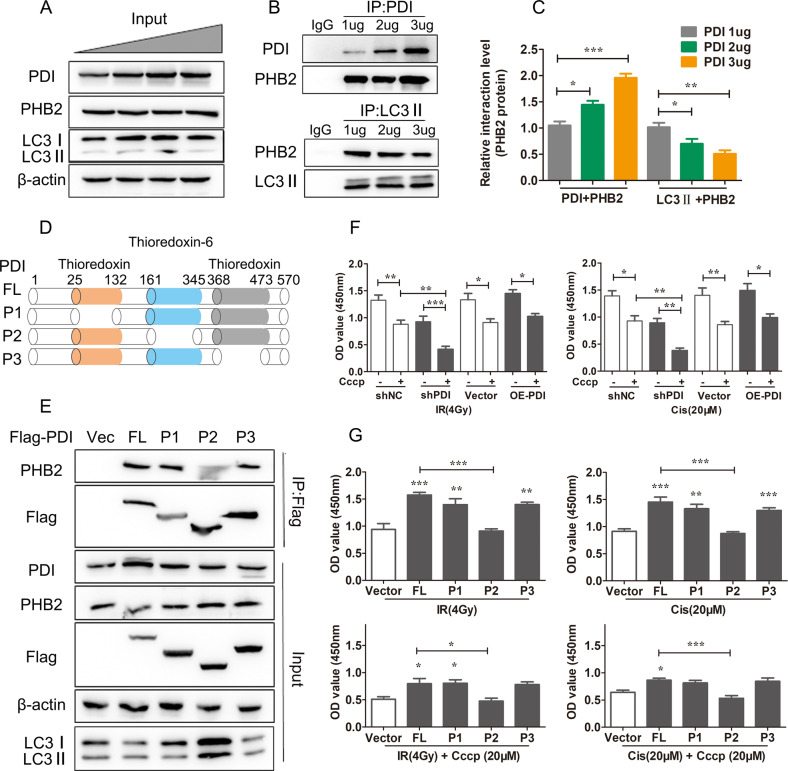
PDI interacts with PHB2 to attenuate autophagic activity. **A**–**C** HCT116 cells were transfected with 1–3 µg PDI-Flag vector (**A**). Immunoprecipitation of PDI by anti-PDI was performed to determine the competitiveness of the interaction (**B**). and the statistical data are shown in (**C**). **D** Schematic diagram of PDI mutant model. **E** HCT116 cells were transfected with PDI (Vector), PDI (WT), PDI (P1), PDI (P2), and PDI (P3) and stimulated with IR (4 Gy). The lysates were subjected to immunoprecipitation with an anti-Flag antibody. The precipitates were analyzed by immunoblotting with PDI, PHB2, Flag, LC3, and ACTIN. **F**, **G** CCK-8 assay analysis of the impact of PDI (Vector), PDI (WT), PDI (P1), PDI (P2), and PDI (P3) on the viability of HCT116 cells after stimulation with IR (4 Gy) or cisplatin (20 μM). Data were pooled from three independent experiments and the results are represented as mean ± SD; *P < 0.05, **P < 0.01, ***P < 0.001.

As PHB2 promotes mitophagy by recruiting LC3II [41], we further examined whether PDI competes with LC3IIto bind PHB2 and inhibits mitophagy. Here, we found that overexpression of PDI can block the interaction between PHB2 and LC3II in cells transfected with a varied dose of PDI expression vector. With the higher expression of PDI, more PHB2 can be pulled down by anti-PDI, and less PHB2 can be pulled down by anti-LC3II (Fig. 7A–C). These results suggest that PDI preferentially binds to PHB2 under the treatment of radiation. We next generated several deletion mutants of PDI to map the domain that interacts with PHB2 (Fig. 7D). As shown in Fig. 7E, the thioredoxin-6 domain of PDI at the 161-345 (P2) but not 25-132 (P1) or 368-473 (P3) is required for its interaction with PHB2. In agreement with these findings, the LC3 levels were increased in cells transfected with P2 after IR treatment compared with cells transfected with P1, P3, and FL. To verify the regulatory effect of PDI-mediated autophagy on cell fate, we used the mitophagy inducer carbonyl cyanide 3-chlorophenylhydrazone (CCCP). The results showed that promoting mitophagy could enhance the decline in cell viability induced by radio/chemo-therapy (Fig. 7F). The cell viability assay also showed that IR/Cis treatment alone or the combined treatment with CCCP could further promote the decline of cell viability in cell transfects P2 plasmid but not P1, P3 and FL plasmids (Fig. 7G). The above results indicate that P2 located in the thioredoxin-6 domain of PDI is an important factor in the interaction between PDI and PHB2 to inhibit autophagy.

## Discussion

**Fig. 8:**
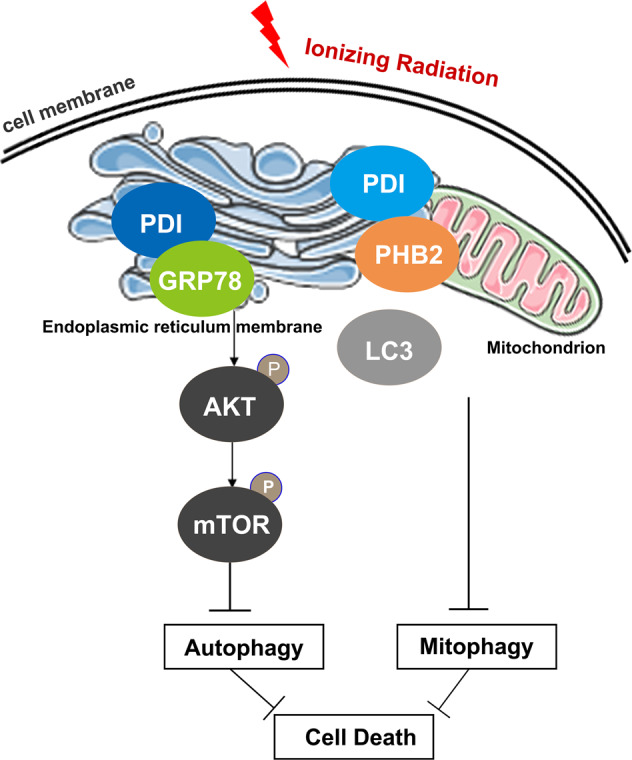
Schematic diagram to show the effect of PDI regulating autophagy in CRC. Under radio/chemo-therapy conditions, PDI interacts with GRP78 to inhibit the degradation of AKT, and eventually activates the mTOR pathway and inhibits autophagy initiation. In parallel, radio/chemo-therapy mediates the transfer of PDI from the ER to mitochondria, and translocated PDI competes with LC3 to bind PHB2 on mitochondria to inhibit mitophagy.

Recent studies have demonstrated that the adaptive autophagy system can regulate radio/chemo-sensitivity of various cancers [5, 42, 43]. However, the potential role of the PDI in regulating autophagy in CRC radio/chemo-resistance is poorly understood. Through a combination of bioinformatics, biological, and clinical studies, we have demonstrated that autophagy-related pathways are enriched and activated in CRC cells and the overexpression of PDI promotes CRC radio/chemo-resistance. Mechanistic studies showed that under radio/chemo-therapy conditions, PDI interacts with GRP78 to inhibit the degradation of Akt, and eventually activates the mTOR pathway and inhibits autophagy initiation. In parallel, radio/chemo-therapy mediates the transfer of PDI from the ER to mitochondria, and translocated PDI competes with LC3 to bind PHB2 on mitochondria to inhibit mitophagy (Fig. 8). This study proposes that promoting autophagy in specific cells, such as cancer cells with low levels of PDI, can cause excessive autophagy, which will increase the sensitivity of radio/chemo-therapy.

Since high expression of PDI was detected in many cancers, including glioblastoma, non-small cell lung cancer, pancreas, multiple myeloma, and various radio/chemo-resistance cell lines, PDI is of particular interest concerning cancer [23, 44]. PDI might be one potential novel KIRC diagnostic and prognostic biomarker at both the mRNA and protein levels [11]. Here, using new generation sequencing methods together with the detection of clinical samples, we also found that PDI is a prognostic biomarker for CRC (Fig. 1) [45]. To our knowledge, previous studies have highlighted the oncogenic role of the PDI family in cancer cells and have established an important role in the quality control of the autophagy system [14, 18]. Some researchers have conjectured that the capacity of PDI to regulate protein degradation pathways and autophagy systems may contribute to the radio/chemo-resistance phenotype [15, 19, 20]. In this study, we found for the first time that the expression level of PDI was significantly correlated with the clinical survival status of patients with colon cancer. There is a positive correlation between cancer grade and PDI expression in colon cancer. PDI promotes the malignant development of tumors.

Our findings displayed a novel mechanism that PDI resulted in the radio/chemo-resistance by reducing the autophagy level. Therefore, the enhancement of autophagy may have a considerable impact on the treatment of radio/chemo-resistance tumors. Consistent with previous results [36], we confirmed that IR/CIS treatment activated the ERS pathway, which enhanced the binding of PDI with GRP78, and then inhibited the degradation of Akt, and finally reduced autophagy signaling [46]. GRP78 facilitates the translocation of mitochondrial stabilization proteins to mitochondria to promote mitochondrial stability and regulate autophagy after ERS [46]. ER-localized GRP78 was also required for multiple protein translocation, and this information indicates that the translocation dependence on GRP78 has an important effect on regulating autophagy [47]. In our study, PDI was widely distributed on the ER membrane and transferred to the mitochondria after radio/chemo-therapy (Fig. 6). Online data analysis and Co-IP assay all observed that GRP78 may function as a PDI substrate protein, and the interaction of PDI and GRP78 was notably promoted by IR/Cis stimulation (Supplementary Fig. S5d and Fig. 5). We suspected that the translocation of PDI to mitochondria may also be dependent on GRP78. HCT116 is a K-Ras-positive cell line and some reports have found that mutations in RAS are associated with autophagy initiation through RAS-MEK-ERK and PI3K-Ak-mTOR signaling pathways [48]. RAS-MEK-ERK pathway plays a central role in tumorigenesis and is a target of anticancer therapy [49]. Based on our sequencing data, although the ERK pathway was activated in HCT116 cells after radiation, AKT-mTOR pathway had the highest enrichment score among KEGG pathways, which implied that it played a dominant role in regulating PDI-mediated autophagy (Supplementary Fig. S5a).

PDI was originally identified as a disulfide isomerase containing two thioredoxin domains that catalyze the formation, breakage, and rearrangement of disulfide bonds, thus radio/chemo-resistance in PDI-overexpression cancer cells was considered to correlate with its enzyme activity [50]. However, we found that inhibition of PDI enzymatic activity had no significant effect on autophagy, suggesting that PDI-regulated autophagy is independent of its enzymatic activity. Besides, the inhibition of the ERS pathway does not block autophagy signaling completely, hence, other pathways are involved in PDI-mediated autophagy. Here, immunofluorescence study indeed found that after IR/Cis treatment, there was a co-localization between PDI and PHB2. The interaction between PDI and PHB2 was further confirmed in vitro. PHB2 is a newly discovered mitochondrial autophagy receptor, which can bind to autophagy membrane-associated protein LC3 through the LC3 interaction region domain to directly mediate autophagy [51]. We also found that the total protein level of PHB2 did not change in PDI knockdown or overexpression cells, but knockdown PDI increased the expression level of PHB2 in purified mitochondria after IR/Cis treatment. Previous studies have shown that proteasome-dependent OMM rupture is required for the interaction between PHB2 and LC3II [41]. We speculated that the depolarization of MMP caused by PDI knockdown may induce the recruitment of the PHB2 to mitochondrial. In the following experiments, it was found that in the presence of activation signaling, translocated PDI interacts with PHB2 through its thioredoxin-6 domain subunit, which competitively blocks the binding of LC3II with PHB2 and subsequently inhibits the mitophagic signaling and further promote cell death induced by radio/chemo-therapy.

In conclusion, the current study discovers a new molecular mechanism of PDI in CRC by regulating ER oxidative stress and the autophagy signaling pathway. Specifically, on the one hand, PDI reduced autophagy through the ERS pathway, and on the other hand, PDI competitive binding to the mitochondrial autophagy receptor PHB2 inhibits autophagy through its thioredoxin-6 domain. Altogether, our studies highlight the essential roles of PDI-dependent autophagy in radio/chemo-resistance. These new molecular insights provide an important contribution to better understand the mechanism of PDI enhancing tumor cell resistance to radio/chemo-therapy.

## Materials and methods

### Patients and tissue specimens

A total of 60 cases of matched CRC and para tumor tissue samples were collected from patients who were diagnosed with CRC from 2016 to 2021 at The Sixth Affiliated Hospital, Sun Yat-sen University (Guangdong, China). Patients were classified according to TNM stags (American Joint Committee on Cancer, 8th edition), and well documented with complete follow-ups for 5 years or until death. According to the established plan approved by the ethics committee of the Sixth Affiliated Hospital of Sun Yat-sen University, samples were obtained with informed consent. The detailed clinic pathological characteristics are given in Table 1.

### Cell cultures, irradiation, and regents

The human cell lines HCT116, HEK293T, A549, HIEC, and MEF cells were kindly provided by Dr Chi Li (University of Louisville, Kentucky, USA). The human cell line LOVO was obtained from the Cell Bank of the Chinese Academy of Sciences (Shanghai, China). Cell lines of MEF were obtained from Craig Thompson (MSKCC, NY, USA). All cells were maintained in DMEM (HyClone, UT, USA), supplemented with 10% fetal bovine serum (Biological Industries, Israel), 100 U/ml penicillin, and 100 µg/ml streptomycin (Gibco, NY, USA), in an incubator with 5% CO_2_ at 37 °C.

Irradiation was carried out in a gamma-ray irradiator (Biobeam GM gamma irradiator, Germany) as previous [52]. Briefly, cells were irradiated by high-energy gamma rays, with a dose rate of 3.27 Gy/min, automatically controlled by the computer. The equipment is maintained and calibrated every year by the manufacturer to ensure the precision of the radiation dose.

Cells were treated with 3-MA (Sigma-Aldrich, Missouri, USA) at a final concentration of 5 mM, 4-PBA (Beyotime, Shanghai, China) 10 mΜ, CQ (Topscience, Shanghai, China) 50 mΜ, Rapa (Beyotime, Shanghai, China) 50 nM, Bac (Sigma-Aldrich, Missouri, USA) 500 mΜ, securinine 10 Μm (Thermo Fisher, CA, USA), Z-VAD-FMK (Sigma-Aldrich, Missouri, USA) 20 μM, Cccp (Sigma-Aldrich, Missouri, USA) 20 μM. MK2206 (Beyotime, Shanghai, China) 1 mΜ, MG132 (Beyotime, Shanghai, China) 10 mΜ, Bafilomycin A1 (Thermo Fisher, CA, USA) 100 nM. All agents were added in DMSO or ddH_2_O with an equal volume of vehicle used to treat control cells (0.1–0.5% DMSO).

### Plasmids and antibodies

PDI-Flag, PHB2-Flag plasmids constructed with pcDNA3.1(+) cloning vector were purchased from GENEWIZ company (Suzhou, China). LC3-His, PDI-Myc, plasmids constructed with pET-28a (+) cloning vector was purchased from Genomeditech Company (Shanghai, China). The antibodies were purchased from Proteintech (IL, USA) as follows: anti-PDI (11245-1-AP), anti-PEPK (20582-1-AP), anti-ATF6 (24169-1-AP), anti-TOM20 (11802-1-AP), anti-TOM40 (18409-1-AP), anti-Calnexin (10427-1-AP), anti-IRE1 (27528-1-AP), anti-PARP (13371-1-AP), anti-AKT (60203-2-Ig), anti-Actin (66009-1-Ig), anti-LC3 (14600-1-AP). The antibodies were purchased from Cell Signaling (MA, USA) as follows: anti-P62 (#39749), anti-PHB2 (#14085), anti-PINK1 (#6946), anti-mTOR (#2972), anti-p-mTOR (#5536), anti-Bcl-2 (#4223), anti-Caspase-7 (#9492), anti-p-AKT (#4060), anti-LC3A/B (#4108). The antibodies were purchased from Santa (CA, USA) as follows: anti-GRP78 (sc-13539), and anti-PHB2 (sc-133094). The antibodies were purchased from Beyotime (Shanghai, China) as follows: anti-FLAG (AF0036), anti-HIS (AF5060), and anti-MYC (AF0033).

### Stable cell line construction

pLKO.1-puro lentiviral vectors expressing shRNAs targeting PDI were purchased from Sigma-Aldrich. All vectors contain a puromycin antibiotic resistance gene for selecting transduced cells. Lentivirus production and transduction were performed as described previously [53]. Briefly, transduced HCT116, A549, and MEF cell populations were selected with 1.5 µg/ml puromycin for 7 days and knockdown efficiency was identified by western blotting. shRNA sequences used in the study are listed in the fellow. PDI-shRNA-1-F: CCGG CGAC AGGA CGGT CATT GATT ACTC GAGT AATC AATG ACCG TCCT CTCG TTTT TG. PDI-shRNA-1-R: AATT CAAA AACG ACAG GACG GTCA TTGA TTAC TCGA GTAA TCAA TGAC CGTC CTGT CG. PDI-shRNA-2-F: CCGG TGAC CACG TACA AGCC CGAA TCTC GAGA TTCG GGCT TGTA CTTG GTCA TTTT TG. PDI-shRNA-2-R: AATT CAAA AATG ACCA AGTA CAAG CCCG AATC TCGA GATT CGGG CTTG TACT TGGT CA. PDI-shRNA-3-F: CCGG GTGT GGTC ACTG CAAA CAGT TCTC GAGA ACTG TTTG CAGT GACC ACAC TTTT TG. PDI-shRNA-3-R: AATT CAAA AAGT GTGG TCAC TGCA AACA GTTC TCGA GAAC TGTT TGCA GTGA CCAC AC.

### Ad-mRFP-GFP-LC3B transfection

Ad-mRFP-GFP-LC3B vectors were transfected according to the manufacturer’s instructions (Beyotime, Shanghai, China), and transfected cells were cultured for 48 h. After transfection with adenoviral vectors and treatments as described in figure legends, cells were washed with PBS and fixed with ice-cold 100% methanol for 30 min. After washing, coverslips were mounted on glass slides and examined using a fluorescence microscope (Olympus, Tokyo, Japan).

### Immunohistochemistry tissues

Tissues fixed with 4% paraformaldehyde in PBS for 60 min. After permeabilization, sections of tissue were stained using PDI antibodies. Images were acquired using an optical microscope or a laser scanning confocal microscope FV1000 (Olympus, Tokyo, Japan) and Leica TCS SP8 (Leica, Tokyo, Japan). PDI immunostaining was evaluated using a semi-quantitative scoring system.

### Transmission electron imaging

Cells cultured on a 12-well incubator slide were fixed in 4% paraformaldehyde for 30 min, treated with 01% Triton X- 100 in PBS for 30 min, and incubated with 3% BSA under RT for 2 h at RT. After overnight incubation with primary antibody at 4 °C, the cells were washed and incubated with secondary antibody under RT for 2 h. DAPI (Sigma-Aldrich, Missouri, USA) was used to stain the nuclei. Images were visualized by an orthostatic two-photon confocal microscope (Nikon, Tokyo, Japan).

### Co-immunoprecipitation and western blot

Extract the cell lysates from the cell lysis buffer and quantify the protein concentration in the lysates using the BCA protein determination kit (Thermo Fisher, CA, USA). Protein samples with 20–60 μg were loaded for IB. For Co-IP samples, equal amounts of protein were incubated with the primary antibody or non-specific immunoglobulin G and protein G-agarose beads (Beyotime, Shanghai, China) at 4 °C with rotation overnight after cell lysis. The beads were collected by centrifugation and washed five times with the RIPA buffer. Immuno-complexes were eluted with 30 µl of SDS sample buffer and heated at 95 °C for 10 min. Cell extracts and immunoprecipitates were separated by SDS–PAGE and transferred onto PVDF membranes (Roche, Basel, Switzerland). Membranes were incubated with primary antibodies followed by secondary antibodies and visualized with ECL plus (Amersham Bioscience, London, UK).

### HIS pull-down assays

LC3-His, PDI-Myc were cloned into the pGEX-6P-1 vector. The construct was transformed into *Escherichia coli* Rosetta2 (DE3) cells. The cells were cultured at 37 °C until the A600 reached 0.6 and then induced with 0.2 mM isopropyl β-d-thiogalactoside (Takara, Tokyo, Japan) for 16 h at 25 °C. The cells were suspended and sonicated. After spinning, the supernatant was incubated with glutathione Sepharose beads (GE Healthcare, Beijing, China) for 12 h. Reduced glutathione (Beyotime, Shanghai, China) was then used to wash the agarose beads and collect the eluent for the indicated experiments. 293T cells were transformed with vector and Flag-PHB2. Cell lysates were immunoprecipitated with anti-Flag beads (Sigma-Aldrich, Missouri, USA). Supernatants (Flag, Flag-PHB2) were mixed with glutathione agarose beads (Thermo Fisher, CA, USA) for 4 h at 4 °C. The beads were washed three times with the corresponding lysis buffer. Finally, the cell lysates were immunoprecipitated with anti-Flag M2 affinity beads (Sigma-Aldrich, Missouri, USA), and then subjected to SDS/PAGE and western blots with antibodies.

### Cell viability, apoptosis, and colony formation assay

The HCT116 and MEF cells were seeded at a density of 2 × 10^3^ per well in 96-well plates. At indicated time post-γ-ray treatment, cell viability was analyzed using the CCK-8 assay (Solarbio, Beijing, China) according to the manufacturer’s instructions. For cell apoptosis analysis, cells were treated according to the instructions provided with the PI apoptosis detection kit (Solarbio, Beijing, China). Apoptotic ratios were analyzed using FlowJo 10.0 Software, both early apoptotic cells and late apoptotic cells were nominated as apoptotic cells. In the colony formation test, 1 × 10^3^ cells were plated in a six-well cell culture plate. After 10 days of growth, the colonies on the plate were fixed with 4% paraformaldehyde, stained with crystal violet, and counted under a ×100 microscope.

### Caspase activity assays

We seeded 5 × 10^3^ PDI overexpressing and knockdown cells and their parental controls in a 96-well plate. Caspase3/7 activity was measured by a Caspase-Glo3/7 Assay (Promega, WI, USA) according to the manufacturer’s protocol.

### Mass spectrometry (MS)

The cells were centrifuged and collected at 12,900 rpm for 15 min at 4 °C. Proteins were immunoprecipitated using anti-PDI or anti-Flag antibodies. Protein A/G PLUS-agarose beads were incubated with proteins for 12 h and washed five times with the lysis buffer. The PDI complexes were eluted from the beads using 0.1 M glycine–HCl. Then, the endogenous PDI complexes were analyzed in linear mode by tandem MS (MS/MS) performed in Shanghai Applied Protein Technology (APTBIO, China). The resulting MS/MS data were processed using the MaxQuant search engine (v.1.5.2.8).

### Cytosol/mitochondria fraction preparation

Cells were lysed by mechanical braking into a detergent-free buffer (0.25 M sucrose, 10 mM HEPES pH 7.5, 1 mM EDTA). Subsequently, according to the manufacturer’s instructions, mitochondria were separated using the cell mitochondrial separation Kit (Beyotime, Shanghai, China).

### Immunofluorescence microscopy

Cells were fixed and permeabilized with ice-cold 100% methanol for 10 min followed by 30 min in PBS 0.1% Triton X-100 and then washed with PBS. The cells were incubated with primary antibody overnight followed by a washing step. The cells were incubated with the secondary antibody for 1 h at RT. Before mounting in fluorescent mounting medium cells were washed three times with PBST. The image was obtained on Olympus fluoview-1000 confocal microscope (Olympus, Tokyo, Japan).

### Statistical analysis

SPSS 17.0 software (IBM, NY, USA) was used for clinical statistical analyses. An unpaired two-tailed Student’s *t*-test was used to compare the two groups with normally distributed data. Pearson’s *χ*^2^ test was applied to analyze the correlation between the expression of PDI and clinicopathological features. The statistical significance of differences between groups was assessed using the GraphPad Prism5 software (San Diego, CA, USA). All data are presented as the mean ± standard error of the mean unless otherwise specified. Student’s *t*-test or one-way ANOVA was used to determine the significance of all quantitative experiments. All experiments were repeated at least three times. *P* < 0.05 was considered significant, statistical significance was defined as *P < 0.05, **P < 0.01, ***P < 0.01, ^#^*P* < 0.05, ^##^*P* < 0.01, ^###^*P* < 0.01.

## Supplementary information

Reproducibility checklist

Supplementary Figure legends

Supplementary Figure S1

Supplementary Figure S2

Supplementary Figure S3

Supplementary Figure S4

Supplementary Figure S5

Supplementary Figure S6

Original Data File

## Data Availability

Other data that support the findings of this study are available from the corresponding author upon reasonable request.
