# Supplementary material for: Protein disulfide isomerase blocks the interaction of LC3II-PHB2 and promotes mTOR signaling to regulate autophagy and radio/chemo-sensitivity
Source: Cell Death Dis. 2022 Oct 6;13(10):851. doi: 10.1038/s41419-022-05302-w (PMC9537141; doi:10.1038/s41419-022-05302-w)
Supplement: Supplementary file 2 — Supplementary Figure legends [file 41419_2022_5302_MOESM2_ESM.docx]

# Supplementary Figure legends

**Figure S1 (a)** Expression of PDI FPKM in colorectal cancer cells. Data are presented as the mean ± SD (***p< 0.001 by Student’s tests). **(b)** TCGA database was used to analyze the expression of PDI mRNA in CRC tissue (T) and normal tissue (N). **(c)** Expression of PDI mRNA in colorectal cancer tissues based on individual cancer stages. **(d)** Transcriptional expression of PDI at a different stage of CRC from the TCGA database. (*P < 0.01, **P < 0.01, ***P < 0.001).

**Figure S2** **(a)** Representative immunoblot analysis of PDI protein in PDI knockdown or overexpression MEF cells. **(b)** Representative immunoblot analysis of PDI protein in PDI knockdown A549 cells. **(c)** CCK-8 assay was used to analyze the effect of PDI knockdown or overexpression on the viability of MEF cells. **(d)** CCK-8 assay was used to analyze the effect of PDI knockdown on the viability of A549 cells. **(e)** The apoptotic cells were assessed by annexin V-FITC/PI staining after being treated with γ-ray (4Gy) or cisplatin (20μM) for 24 h on the A549 cells, flowjo software performs data statistics. **(f-h)** Caspase3/7 kit was used to detect the effect of knockdown or overexpression of PDI combined with γ-ray (4Gy) or cisplatin (20μM) for 24 h on the apoptosis of MEF and A549 cells. **(i)** Colony formation assay showing the effects of PDI knockdown or overexpression on HCT116 cell growth after being treatment with γ-ray (4 Gy)/Cisplatin (20 μM) for 24. **(j)** Statistical results of experimental data on clonal survival of HCT116 cells. Data of at least 3 independent experiments performed in duplicate are presented as mean ± SEM. shNC: shRNA control, shPDI#1/2/3: shRNA-1/2/3 targeting PDI, Vector: control plasmid, OE-PDI: PDI overexpression plasmid. *P < 0.01, **P < 0.01, ***P < 0.001 compared with shNC. ^##^P < 0.01, ^###^P < 0.001 compared with control vector plasmid transfected cells.

**Figure S3** (**a)** Representative immunoblot analysis showing the levels of P62 and LC3 proteins in PDI knockdown A549 cells treated with γ-ray (4Gy) irradiation for 24 h.

**Figure S4** (**a-d)** MEF cells were pretreated with or without 10 µM CQ (a) or 5 mM 3-MA (b) for 2 h, then treated with cisplatin (20 μM) for 24 h. LC3-II accumulation was measured using WB. CCK-8 assay analysis of the impact of cell viability (c-d). **(e-h)** MEF cells treated with cisplatin (20 μM) for 24 h, after that treated with 10 µM rapamycin (e) or EBSS (f). LC3-II accumulation was measured using WB, CCK-8 assay analyzes of the impact of cell viability (g-h). **(i-l)** MEF cells were pre-treated with 500 mM bac (i) or 20 µM securinine (j) for 24 h then treated with cisplatin (20 μM) for 24 h. LC3-II accumulation was measured using WB, CCK-8 assay analyzes of the impact of cell viability (k-l). Data of 3 independent experiments are presented as mean ± SEM. *P < 0.05, **P < 0.01, ***P < 0.01 compared with control.

**Figure S5 (a)** Analysis of autophagy-related KEGG pathway in HCT116 cells sequencing data. **(b)** Representative immunoblot analysis showing the levels of ERS pathway-related proteins GRP78, PERK or AKT, mTOR proteins in PDI knockdown A549 cells treated with γ-ray (4Gy) irradiation for 24 h. **(c)** Representative immunoblot analysis showing the levels of ULK1 proteins in PDI knockdown MEF cells treated with γ-ray (4 Gy) irradiation or cisplatin (20 μM) for 24 h. **(d)** Protein–protein interaction prediction using the PrePPI database revealed that PDI (P4HB) interacts with GRP78 (HSPA5). **(e)** Fluorescence microscope analysis of co-localization of PDI (red) and GRP78 (green) in HCT116 cells after 24 h of treatment with γ-ray (4 Gy) irradiation or cisplatin (20 μM). Blue DAPI staining was used to stain the cell nucleus. Scale bar = 25 μm. **(f)** The colocalization of PDI and GRP78 in HCT116 cells was detected by immunofluorescence. Data of 3 independent experiments are presented as mean ± SEM. **P < 0.01, ***P < 0.01 compared with control.

**Figure S6** **(a)** PDI binding proteins isolated by Co-IP from HCT116 cell lysate were visualized by coomassie blue staining. The existence of PHB2 in the co-precipitated complexes was confirmed by LC-MS. **(b-c)** The correlative analysis of PHB2 and PDI/LC3 in the human CRC datasets (GSE27262) from the GEO database was assessed by Pearson’s correlation test. **(d-e)** Representative immunoblot analysis PHB2 protein levels in MEF cells after 24 h of treatment with IR (4 Gy) or cisplatin (20 μM).
