# Supplementary figures and images for: Protein disulfide isomerase blocks the interaction of LC3II-PHB2 and promotes mTOR signaling to regulate autophagy and radio/chemo-sensitivity
Source: Cell Death Dis. 2022 Oct 6;13(10):851. doi: 10.1038/s41419-022-05302-w (PMC9537141; doi:10.1038/s41419-022-05302-w)

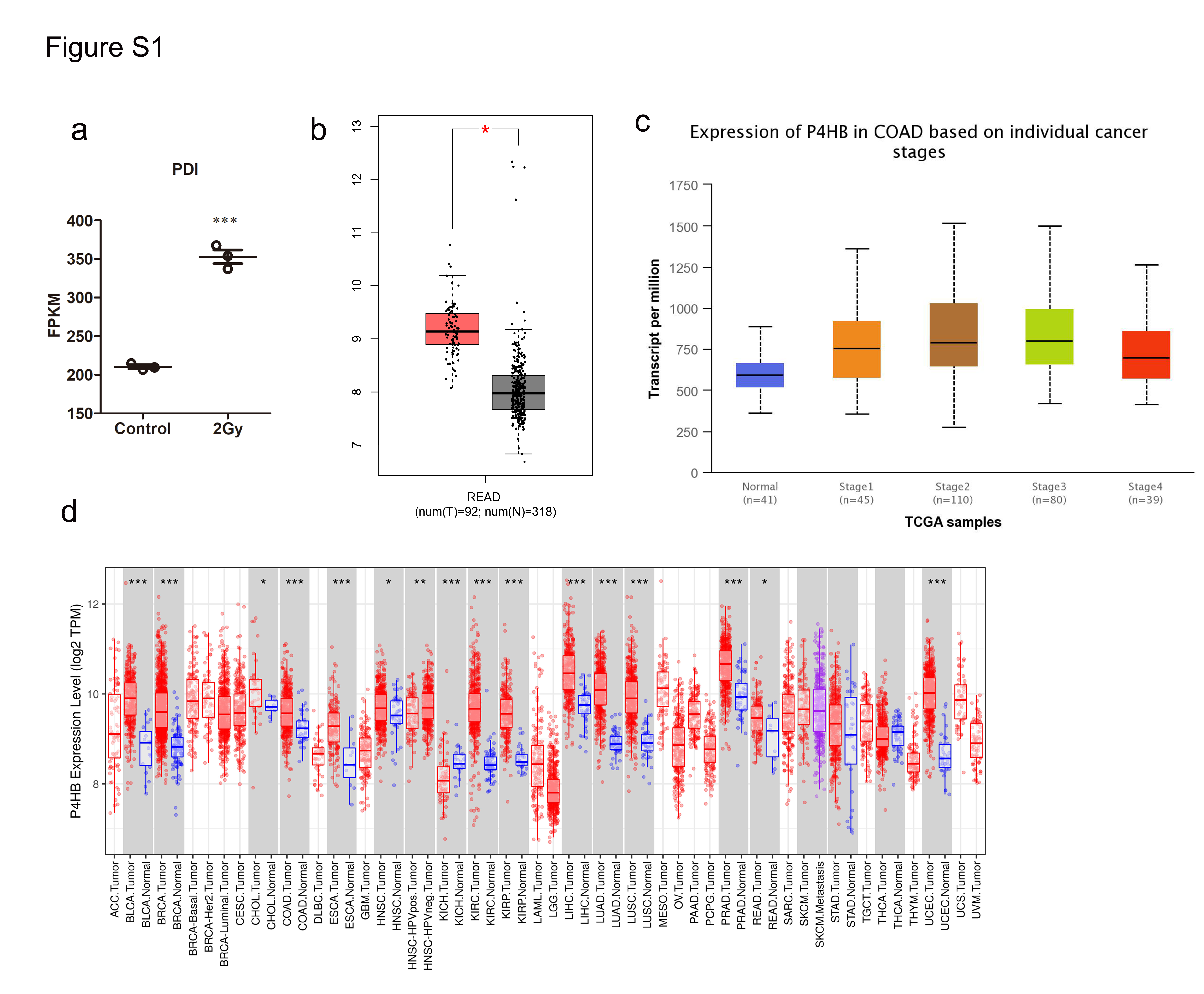

Supplement: Supplementary file 3 — Supplementary Figure S1 [file 41419_2022_5302_MOESM3_ESM.png]

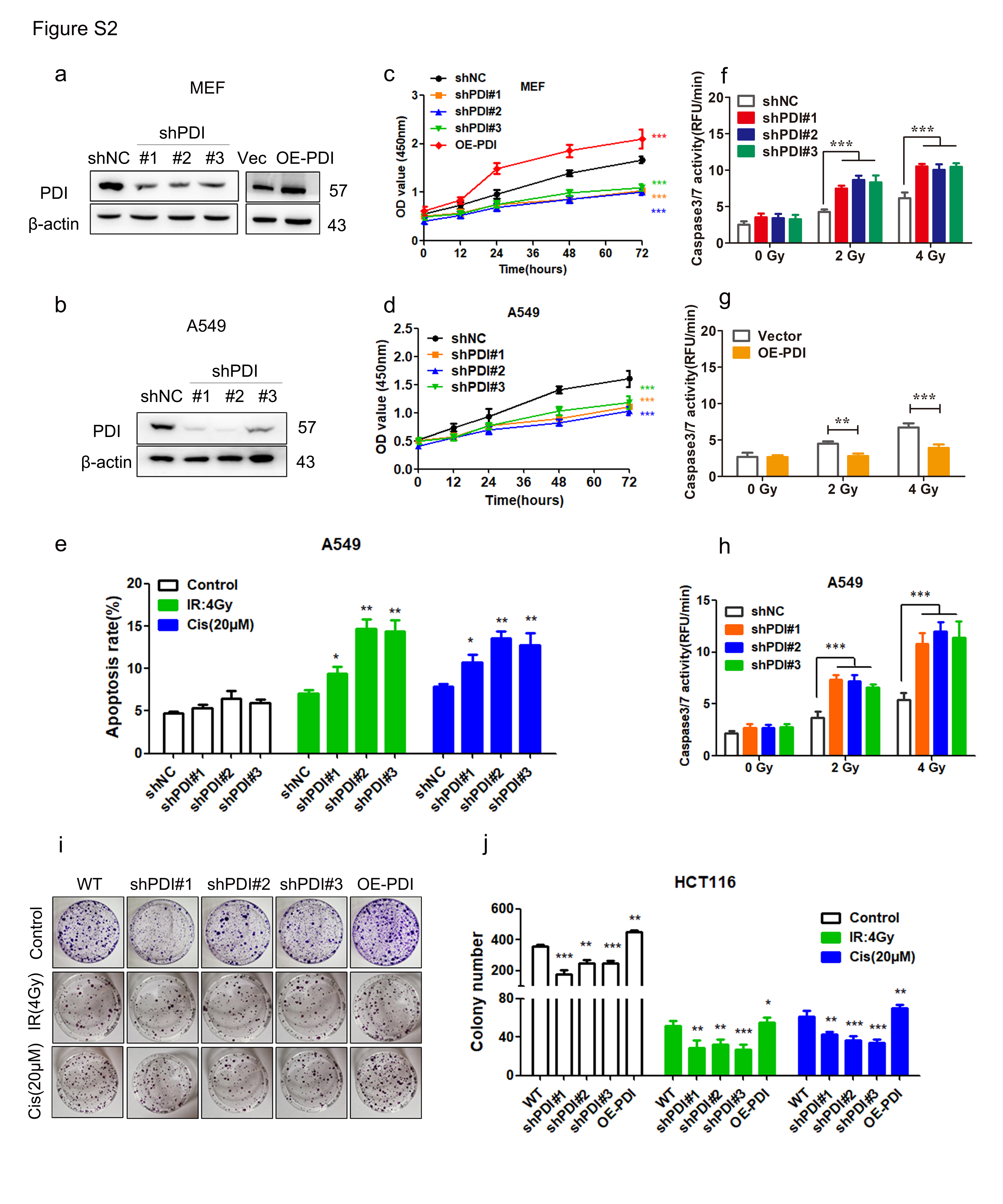

Supplement: Supplementary file 4 — Supplementary Figure S2 [file 41419_2022_5302_MOESM4_ESM.png]

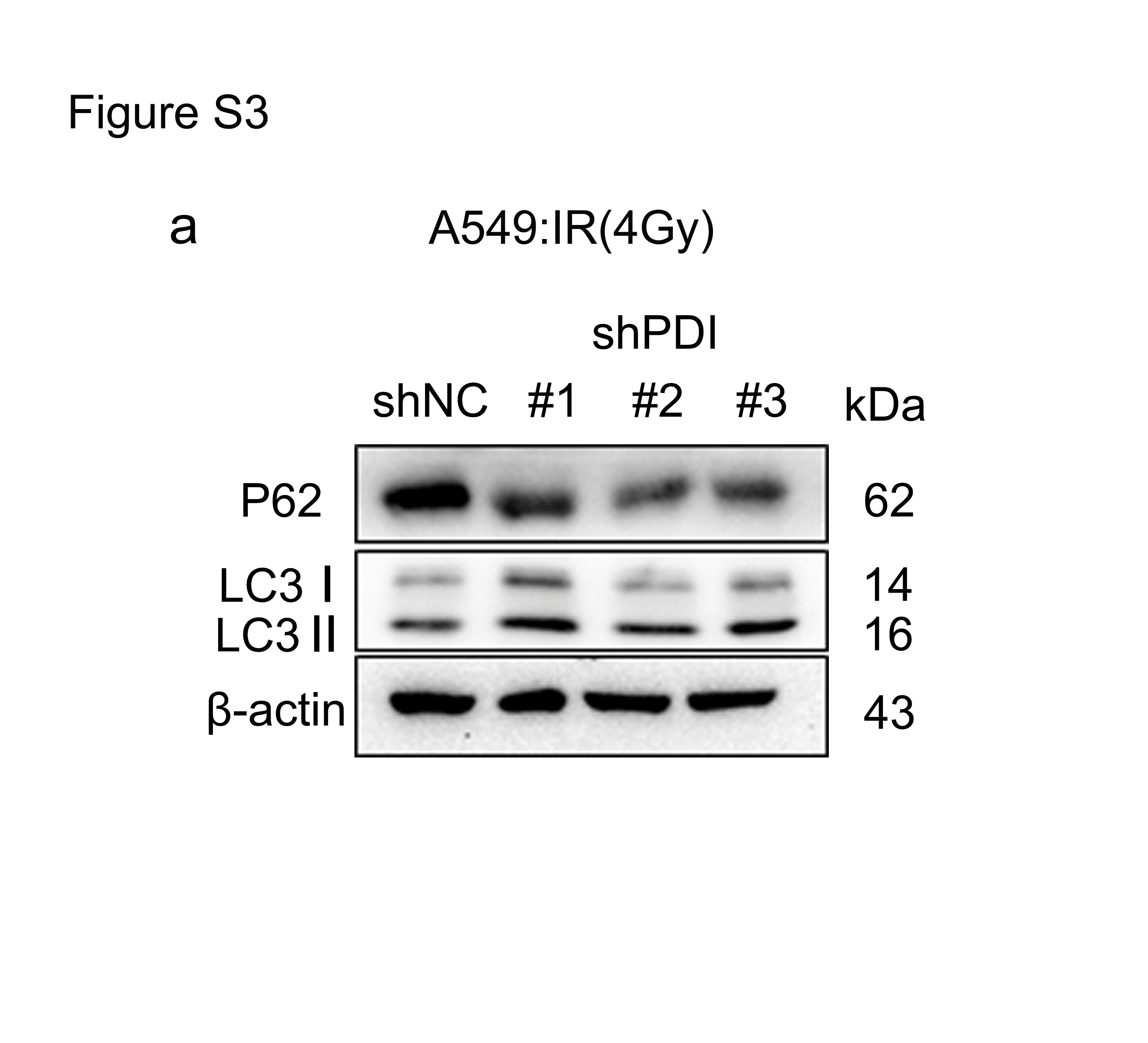

Supplement: Supplementary file 5 — Supplementary Figure S3 [file 41419_2022_5302_MOESM5_ESM.png]

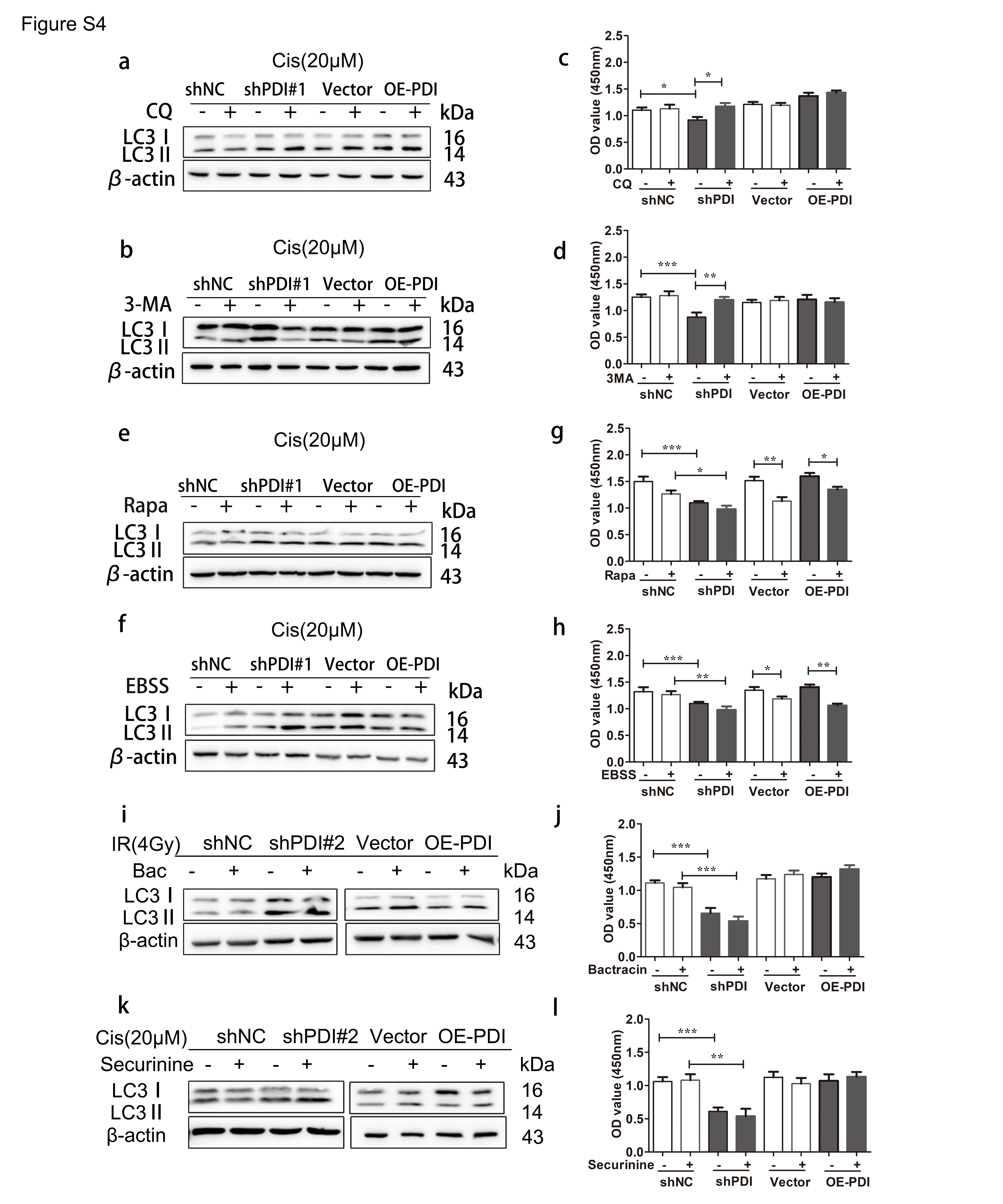

Supplement: Supplementary file 6 — Supplementary Figure S4 [file 41419_2022_5302_MOESM6_ESM.png]

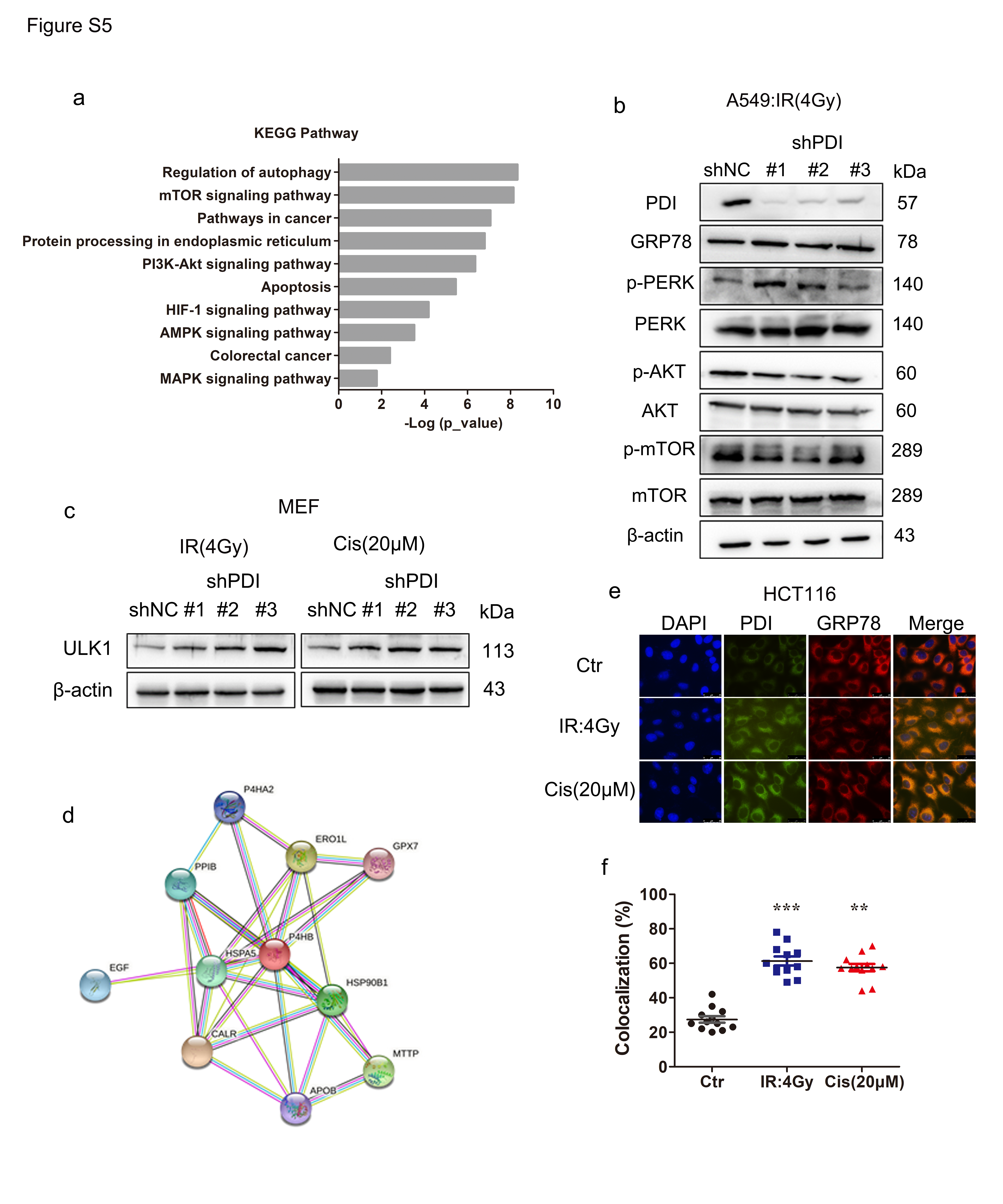

Supplement: Supplementary file 7 — Supplementary Figure S5 [file 41419_2022_5302_MOESM7_ESM.png]

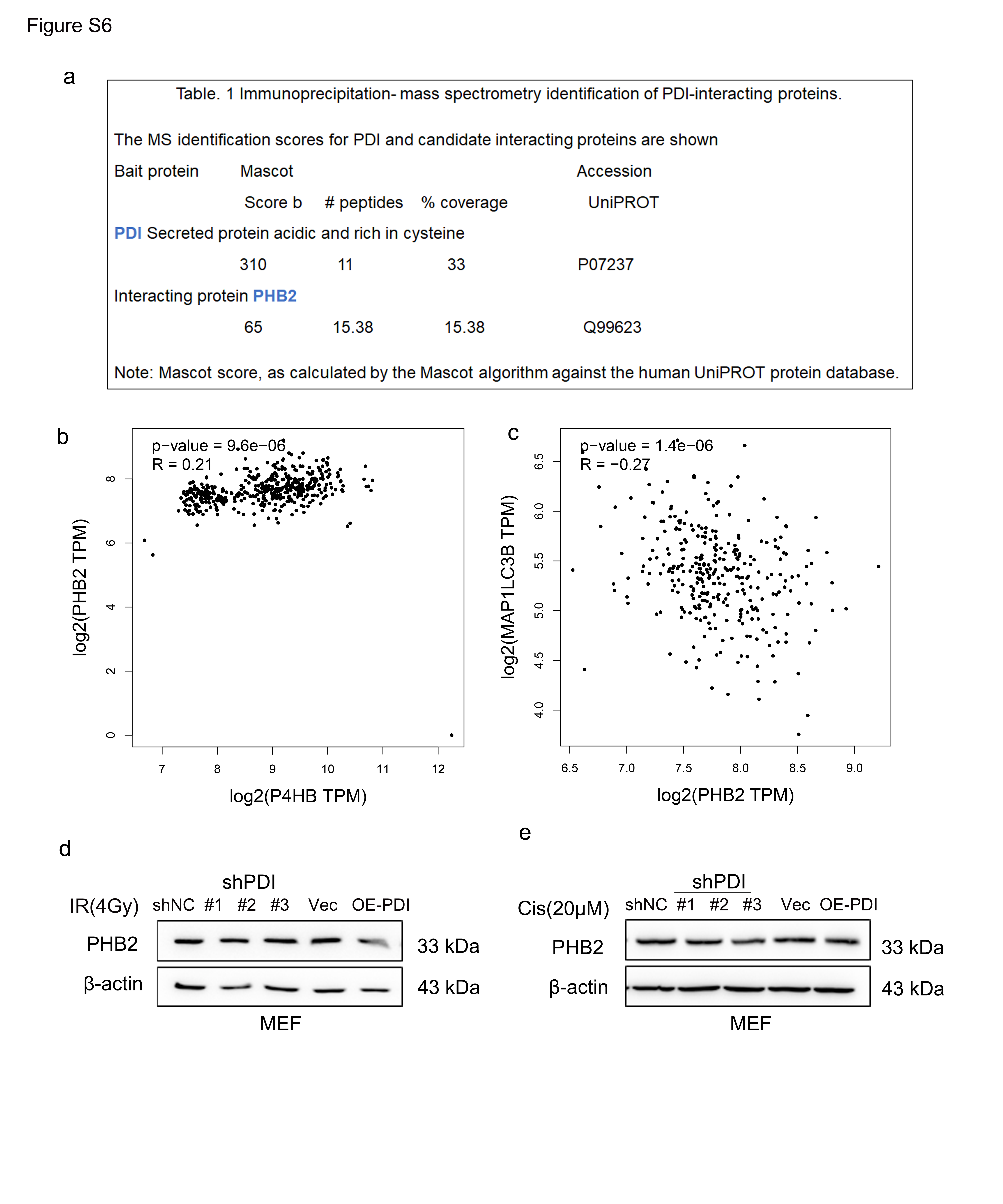

Supplement: Supplementary file 8 — Supplementary Figure S6 [file 41419_2022_5302_MOESM8_ESM.png]
