# Supplementary material for: Protein disulfide isomerase blocks the interaction of LC3II-PHB2 and promotes mTOR signaling to regulate autophagy and radio/chemo-sensitivity
Source: Cell Death Dis. 2022 Oct 6;13(10):851. doi: 10.1038/s41419-022-05302-w (PMC9537141; doi:10.1038/s41419-022-05302-w)
Supplement: Supplementary file 9 — Original Data File [file 41419_2022_5302_MOESM9_ESM.docx]

**Figure 1**

**PDI β-atin**

**



**

**PDI β-atin**

**
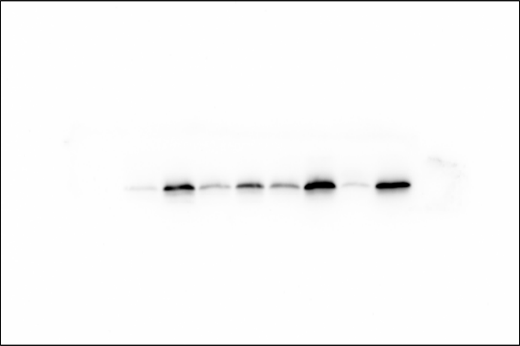

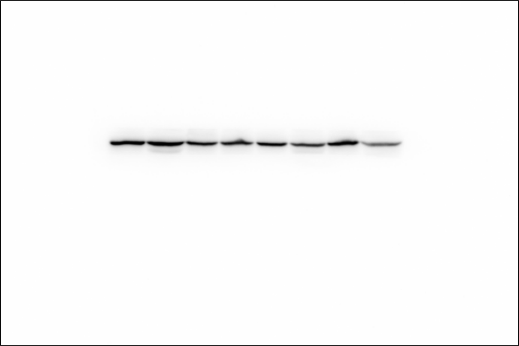
**

**PDI β-atin**

**
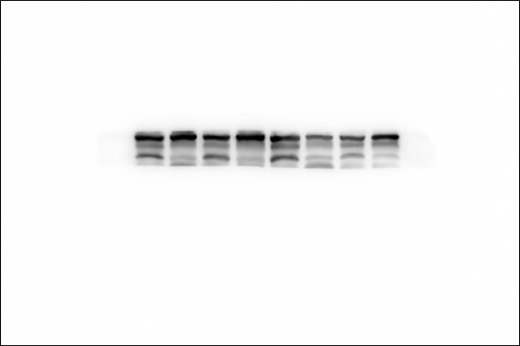

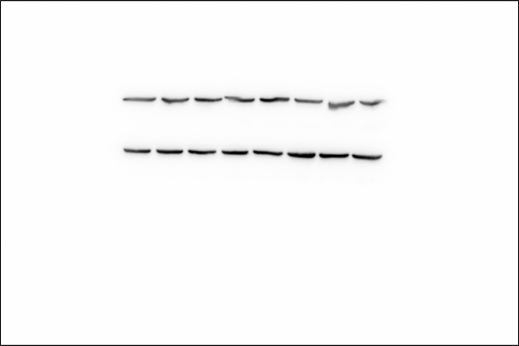
**

**PDI β-atin**

**
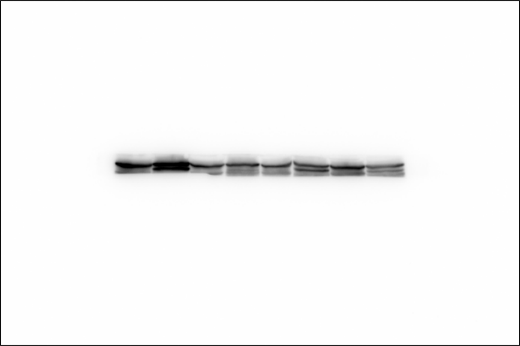

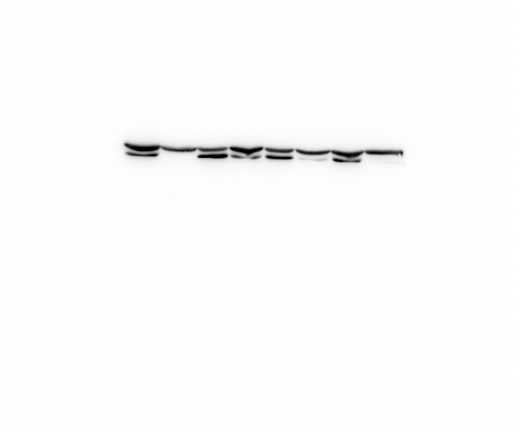
**

**PDI β-atin**

**
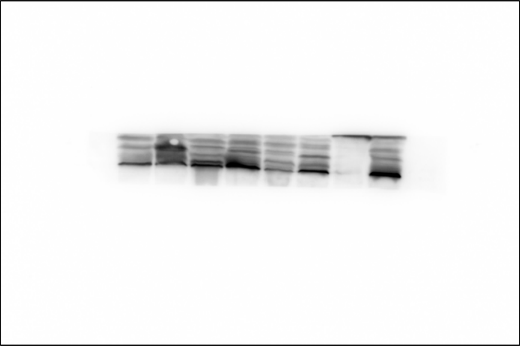

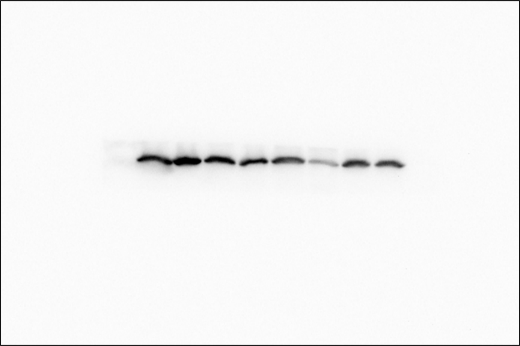
**

**PDI β-atin**

**
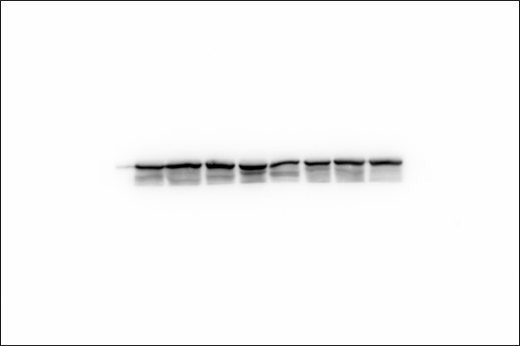

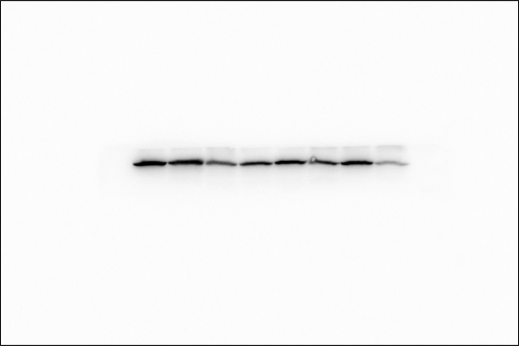
**

**PDI β-atin**

**
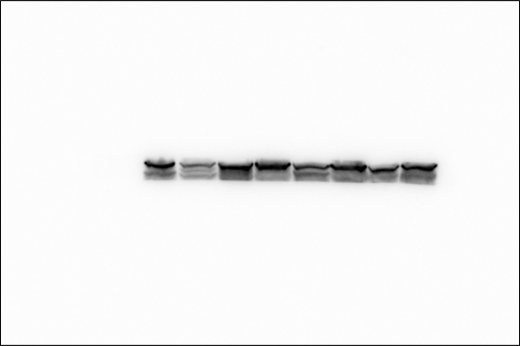

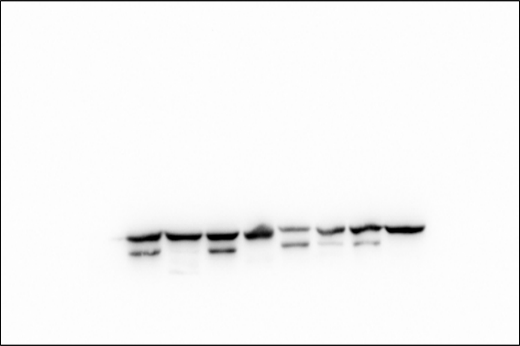
**

**Figure 2**

**PDI β-atin**

**
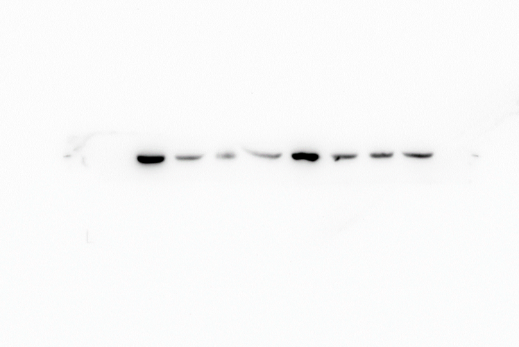

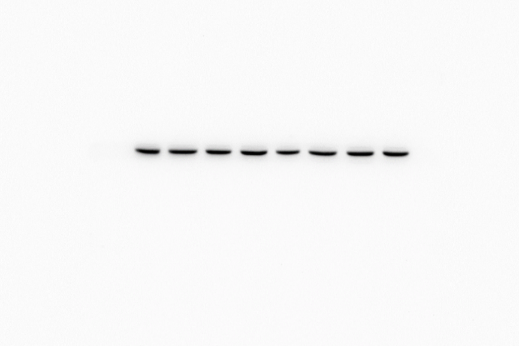
**

**PDI β-atin**

**
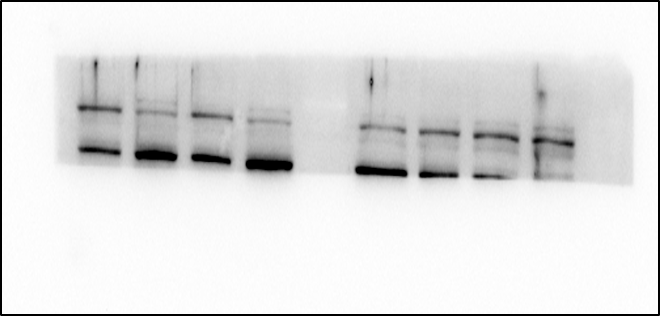

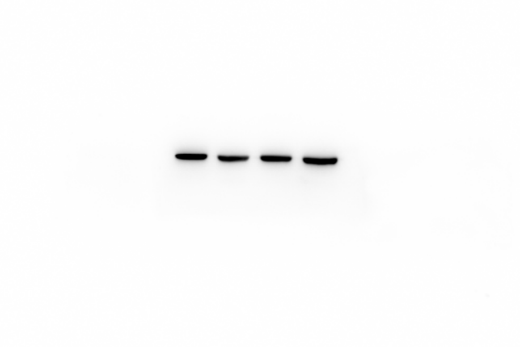
**

**Figure 3**

**P62 β-atin**

**
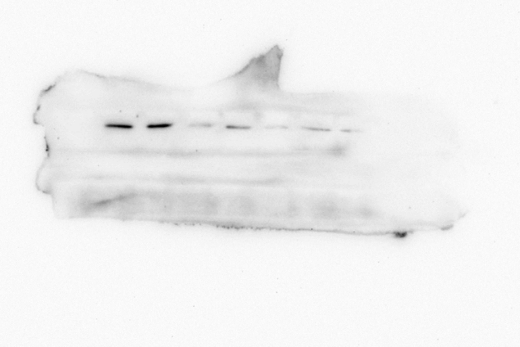

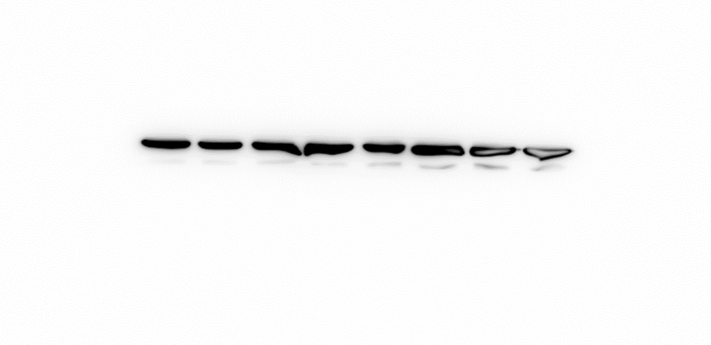
**

**LC3 β-atin**

**
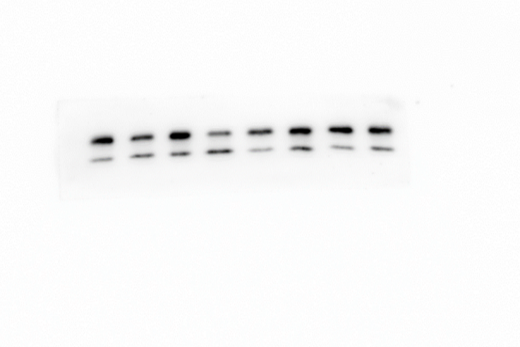

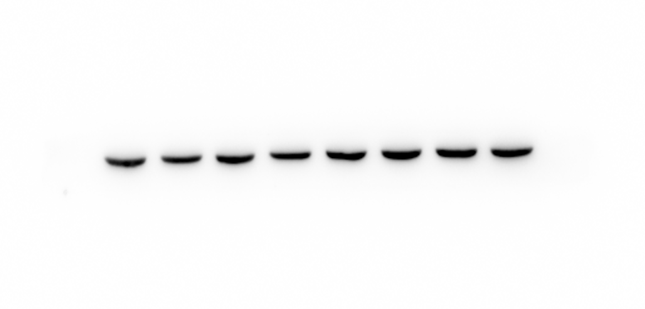
**

**LC3 β-atin**

**
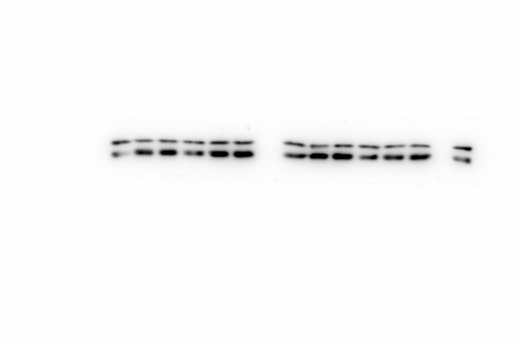

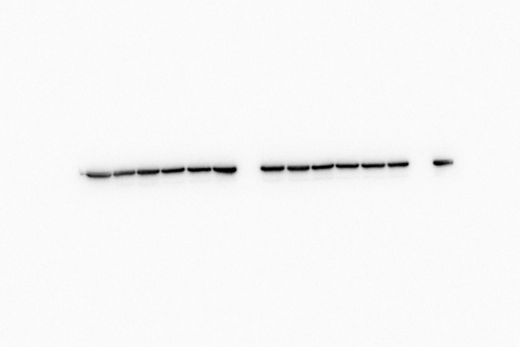
**

**Figure 4**

**LC3 β-atin**

**
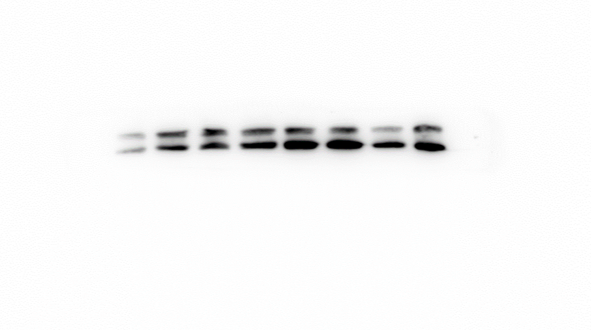

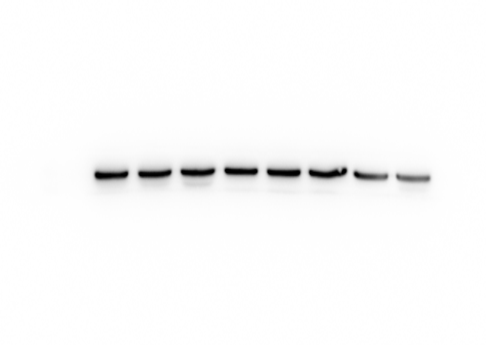
**

**LC3 β-atin**

**
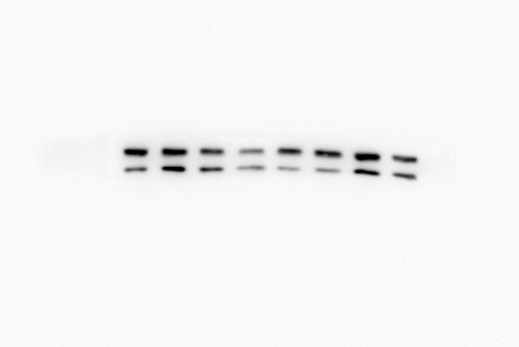

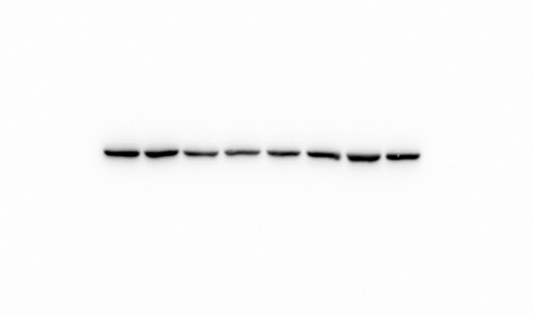
**

**LC3 β-atin**

**
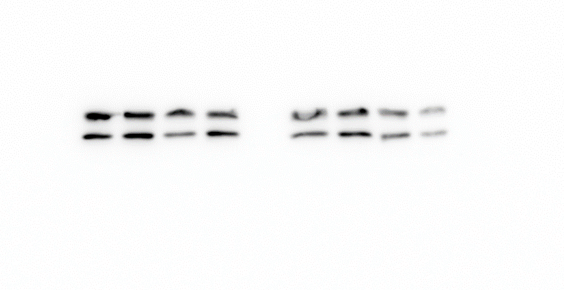

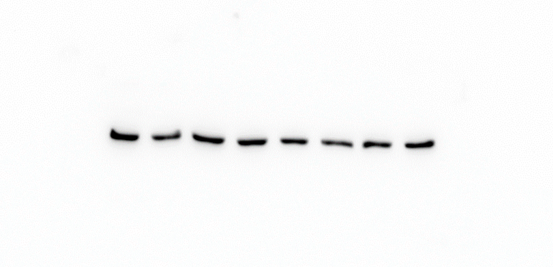
**

**LC3 β-atin**

**
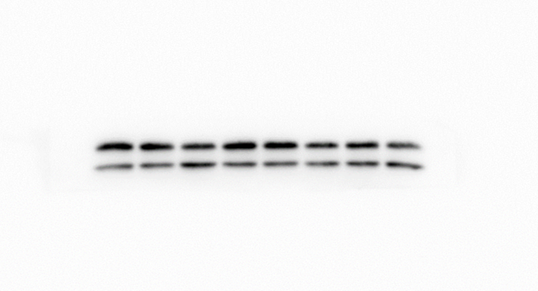

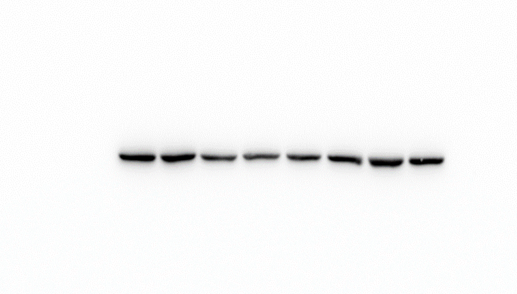
**

**LC3 β-atin**

**
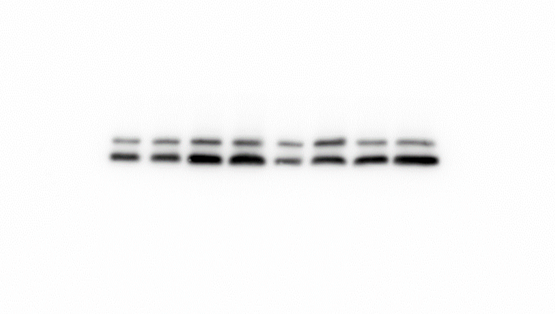

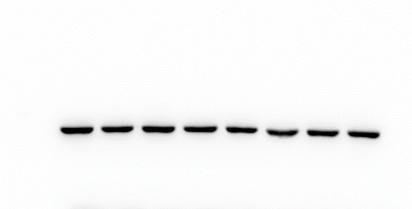
**

**LC3 β-atin**

**
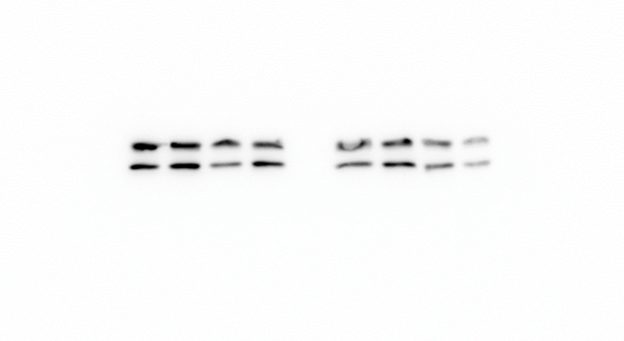

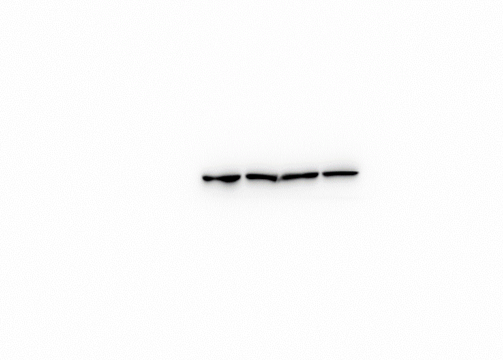
**

**LC3 β-atin**

**
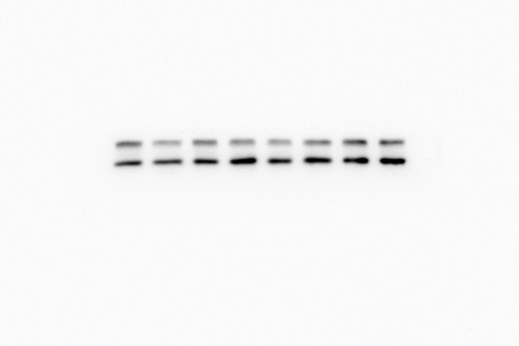

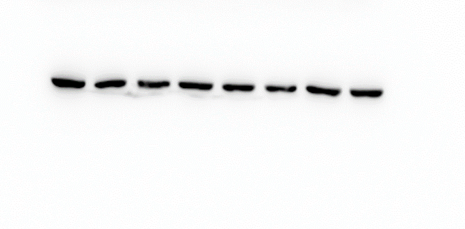
**

**LC3**

**
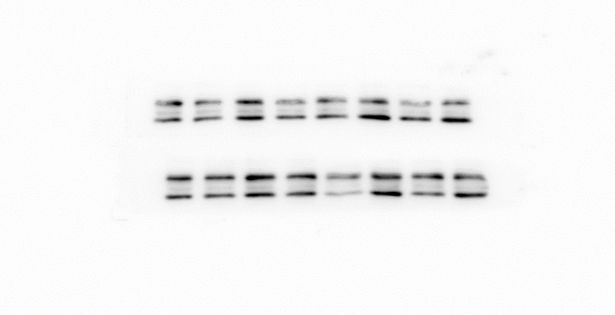
**

**Figure 5**

**β-atin GRP78**

**
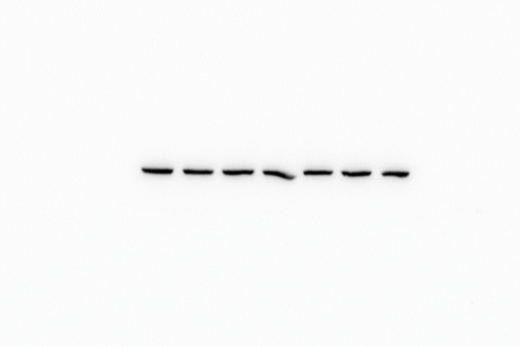

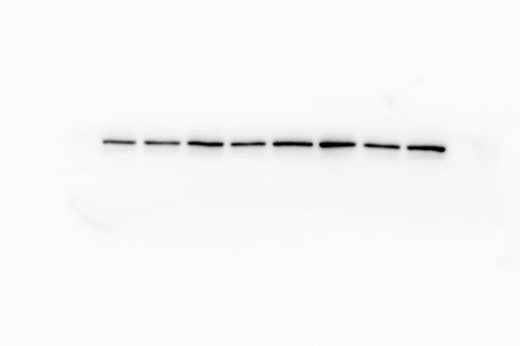
**

**p-AKT PERK**

**
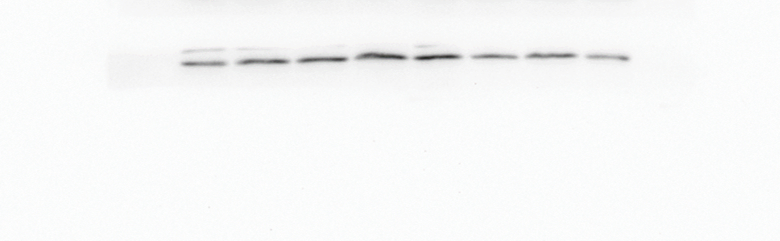

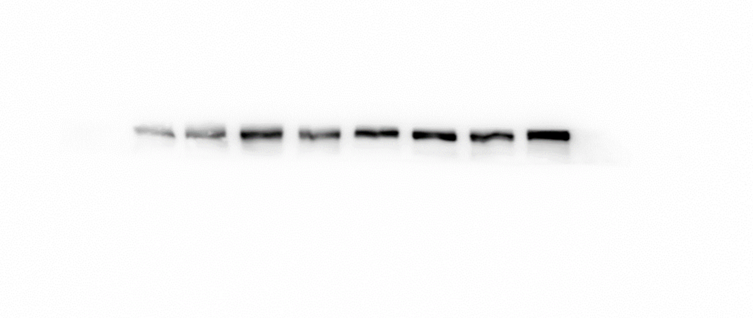
**

**p-mTOR p-PERK**

**
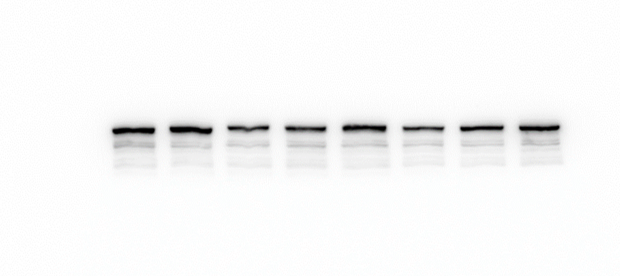

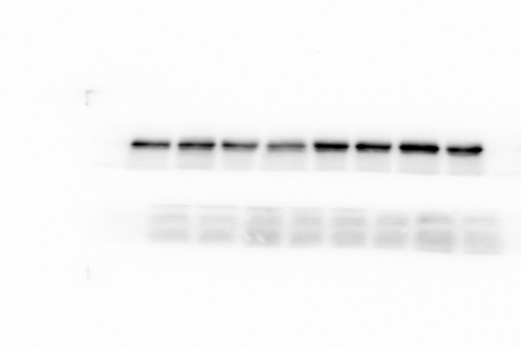
**

**AKT mTOR**

**
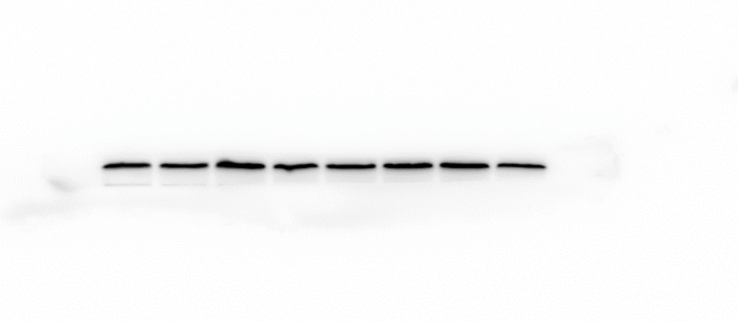

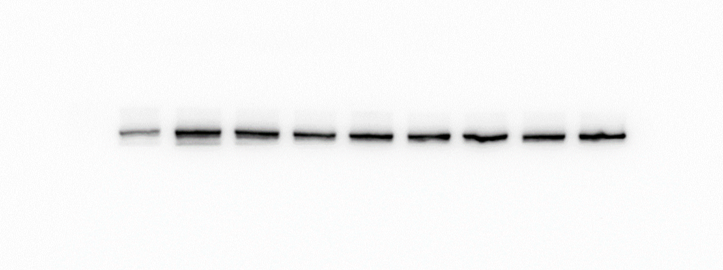
**

**β-atin GRP78**

**
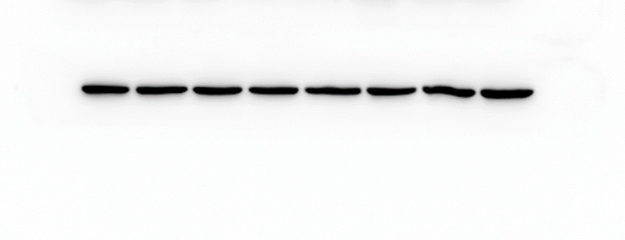

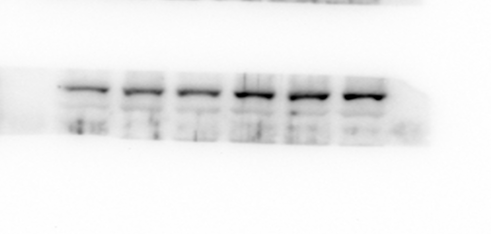
**

**p-AKT PERK**

**
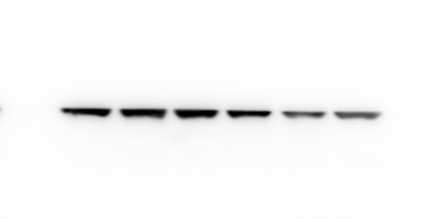

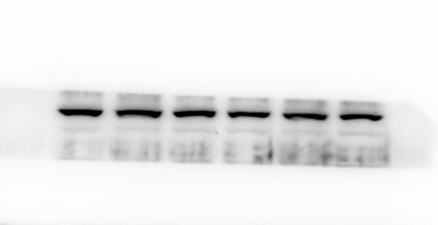
**

**p-mTOR p-PERK**

**
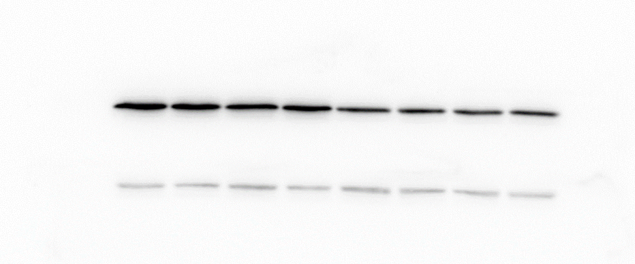

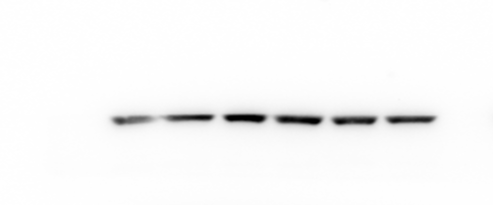
**

**AKT mTOR**

**
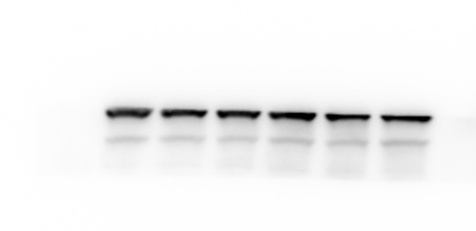

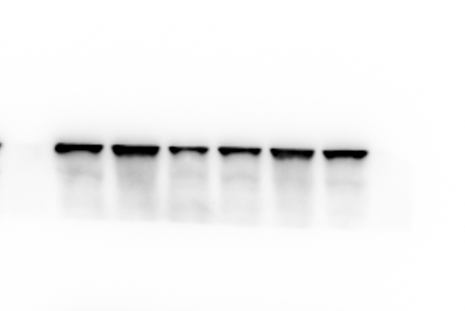
**

**GRP78 PDI**

**
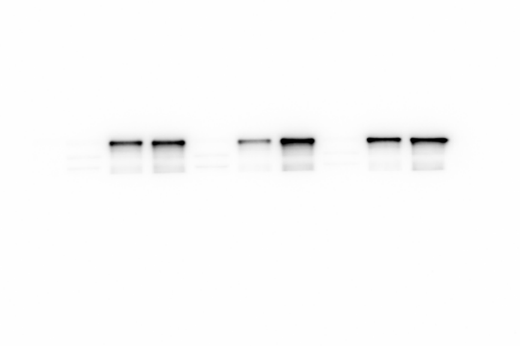

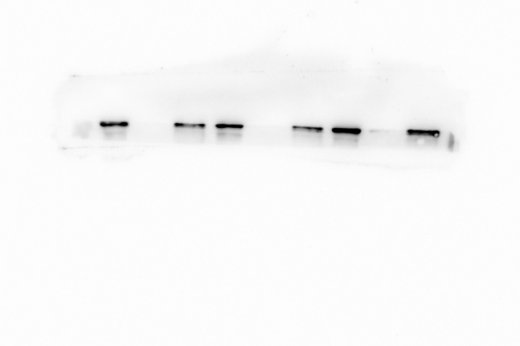
**

**GRP78 PDI**

**



**

**GRP78 PDI**

**
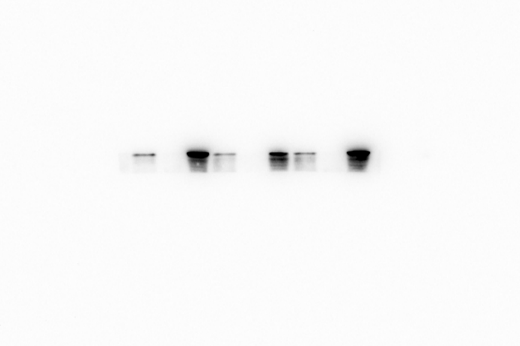

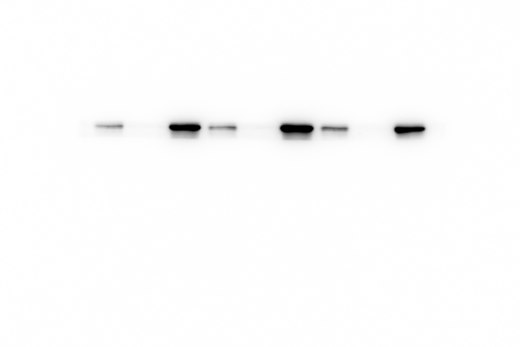
**

**GRP78 PDI**

**
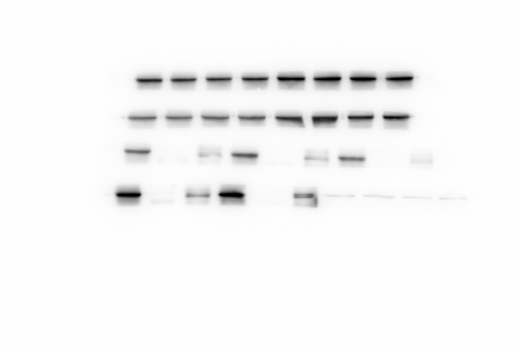

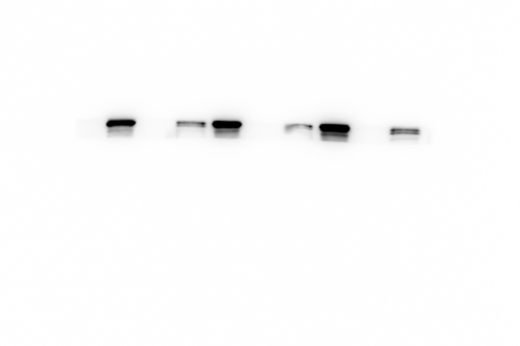
**

**β-atin LC3**

**
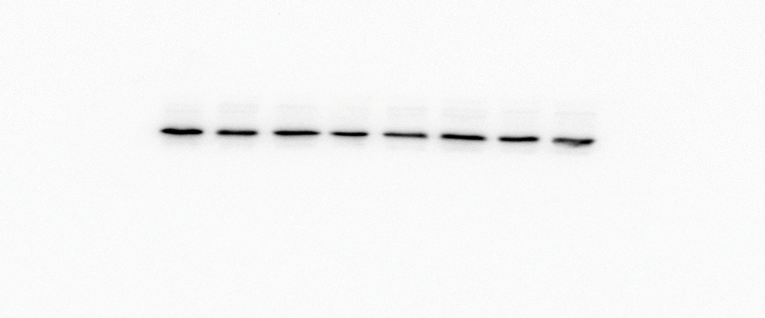

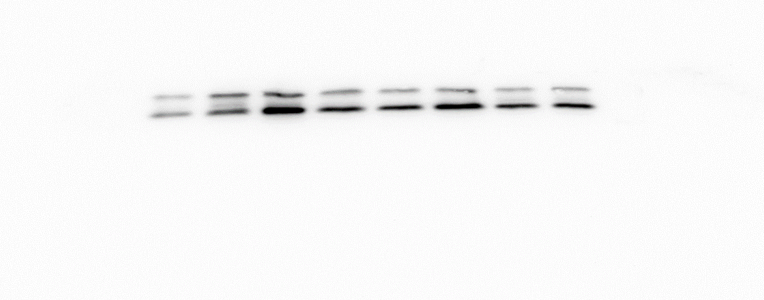
**

**β-atin LC3**

**
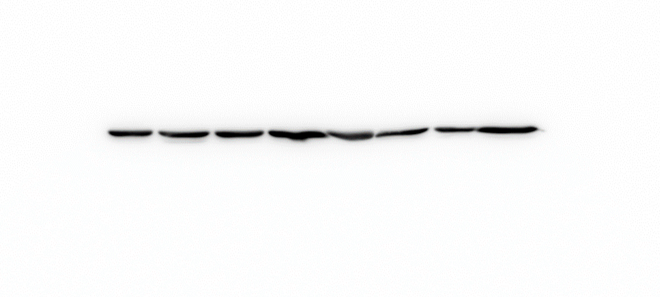

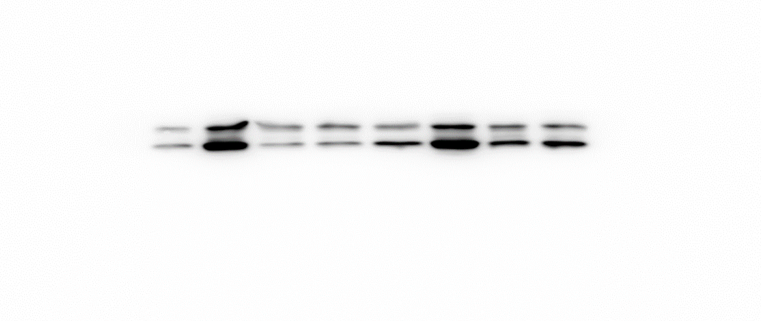
**

**β-atin**

**
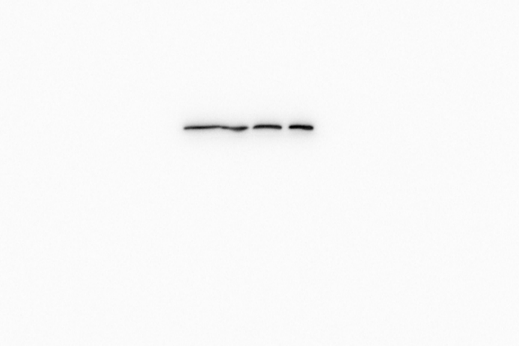
**

**Figure 6**

**PDI PHB2**

**
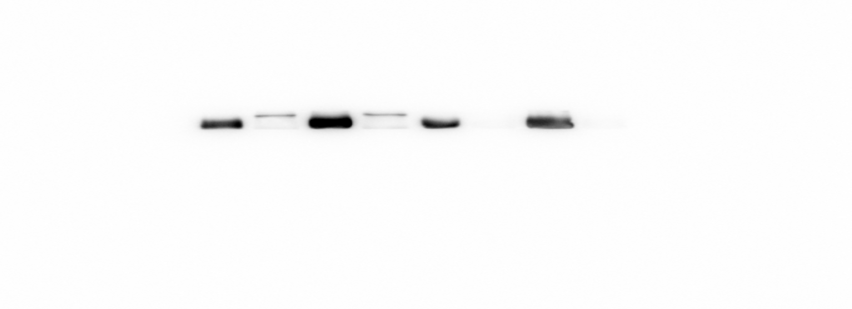

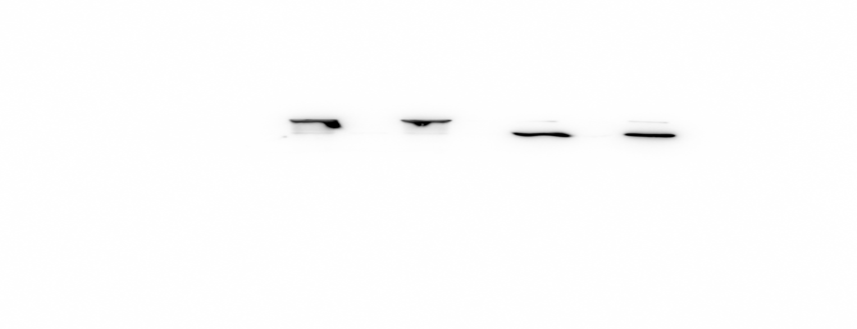
**

**β-atin PDI**

**
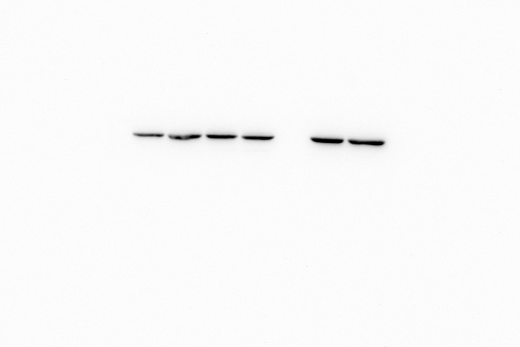

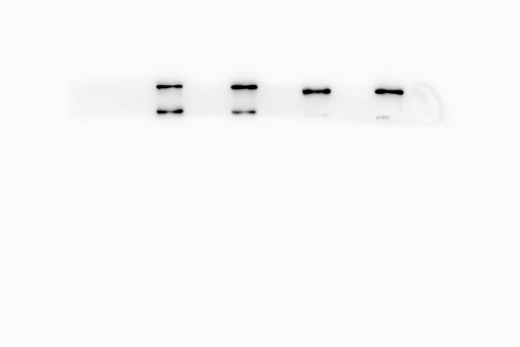
**

**PHB2 β-atin**

**
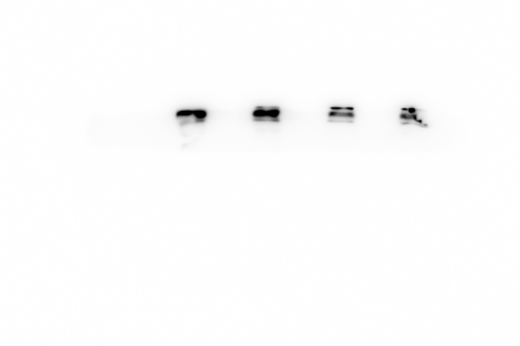

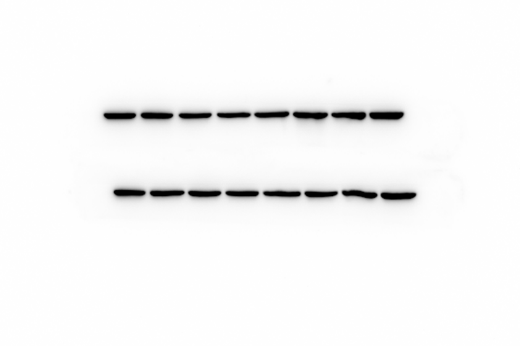
**

**PDI PHB2**

**
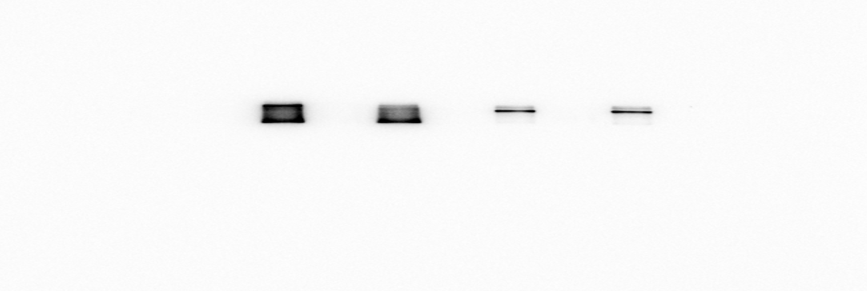

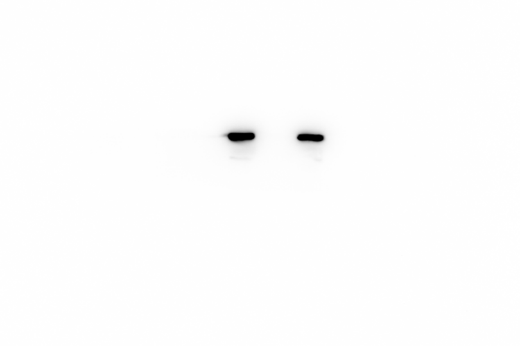
**

**β-atin PHB2**

**
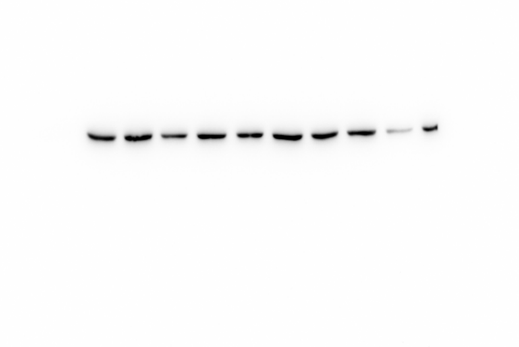

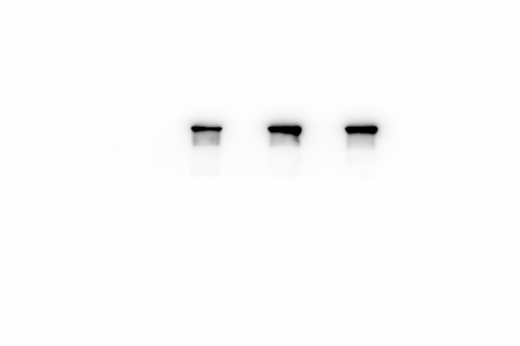
**

**PDI PDI**

**
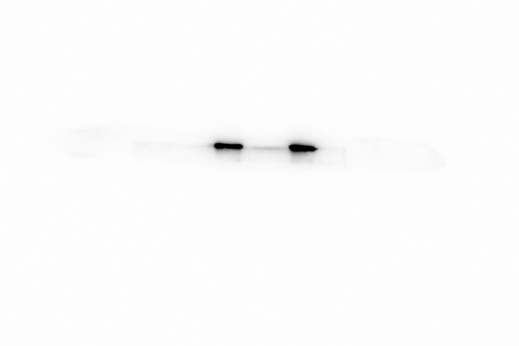

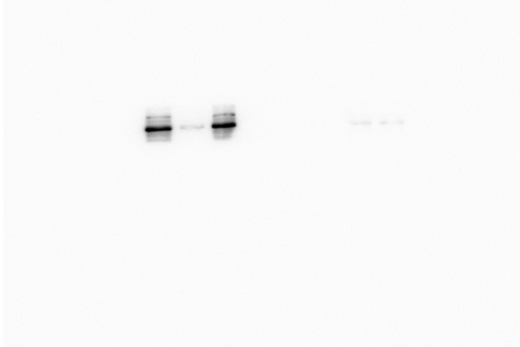
**

**PDI β-atin**

**
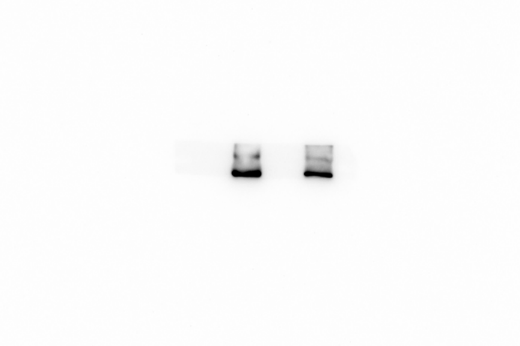

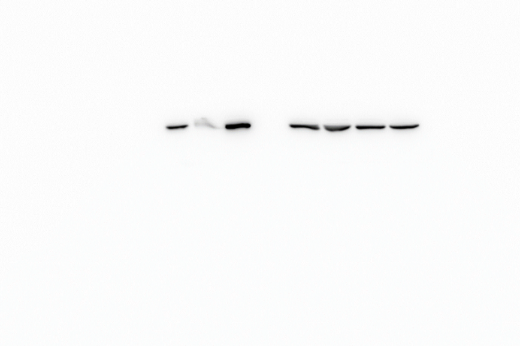
**

**PDI**

**
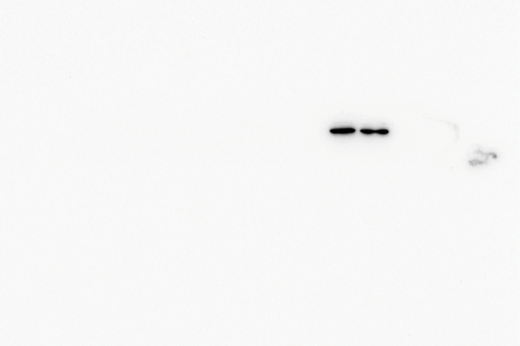

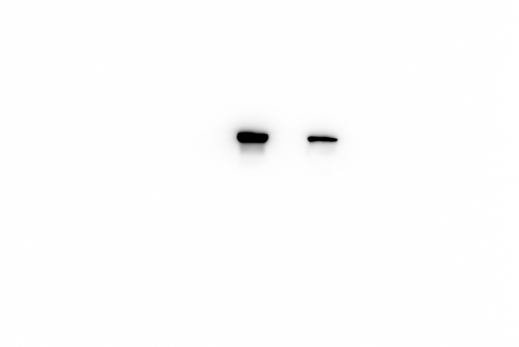
**

**PHB2 PHB2**

**
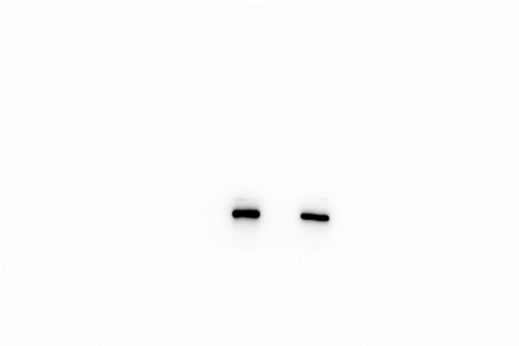

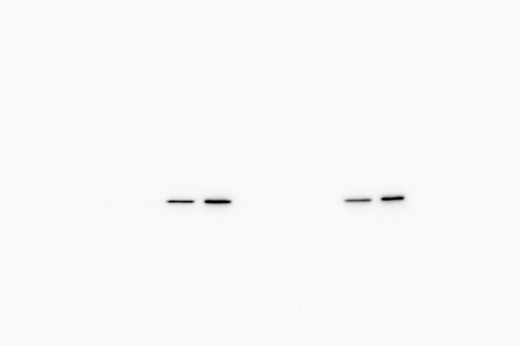
**

**PINK1 PRKN**

**
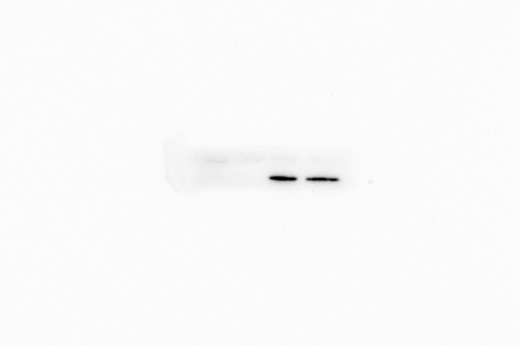

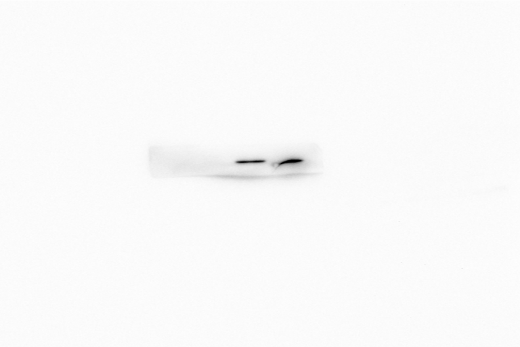
**

**TOM40 TOM40**

**
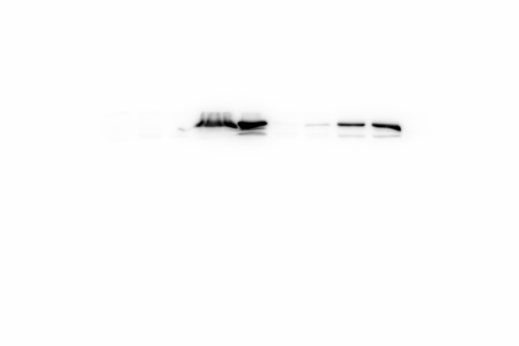

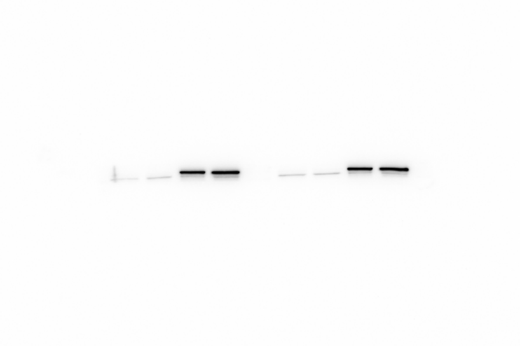
**

**PINK1 PRKN**

**
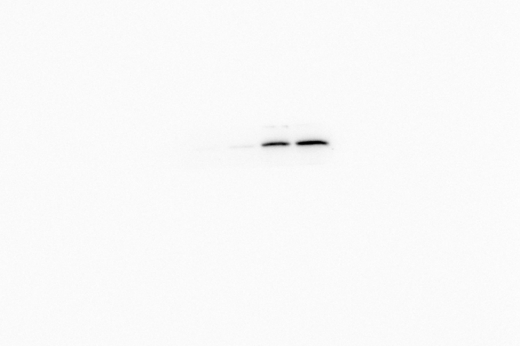

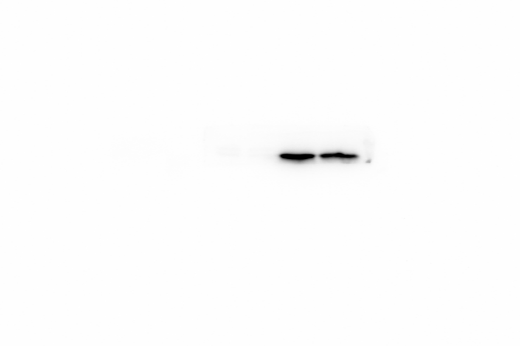
**

**Figure 7**

**PDI β-atin**

**
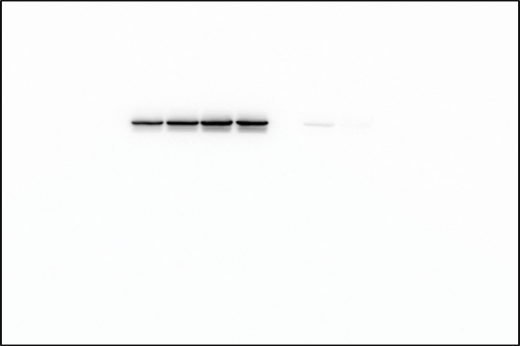

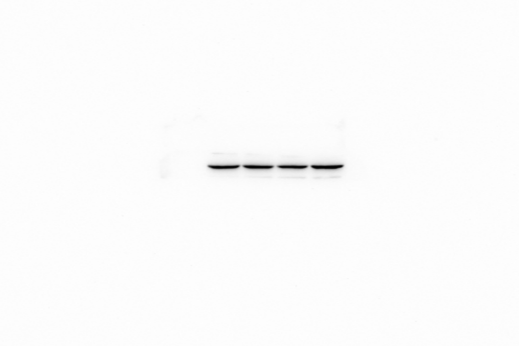
**

**LC3 PHB2**

**
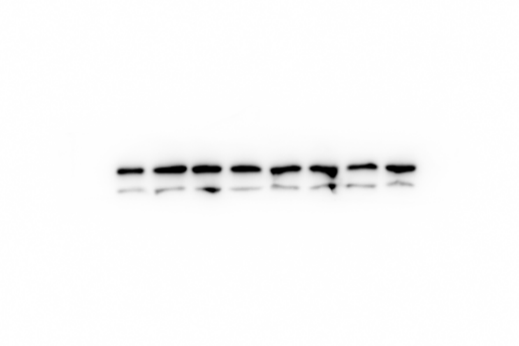

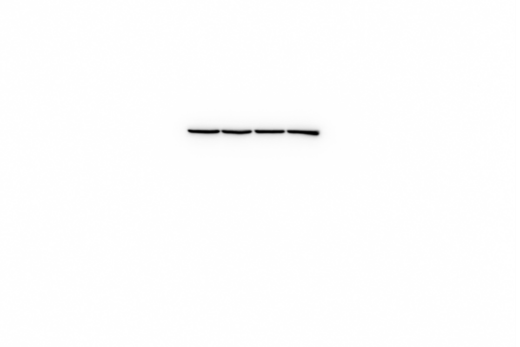
**

**LC3 LC3**

**
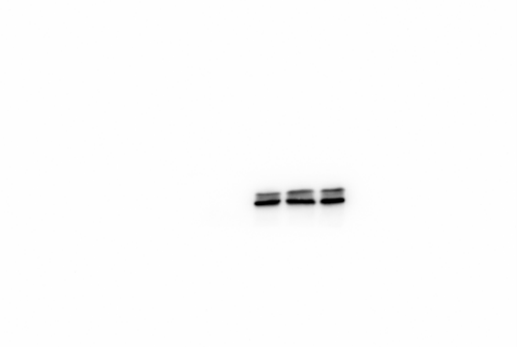

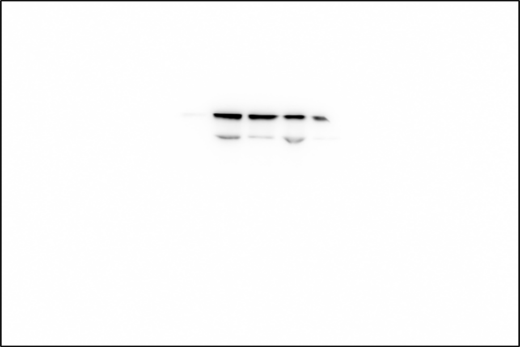
**

**PHB3 FLAG**

**
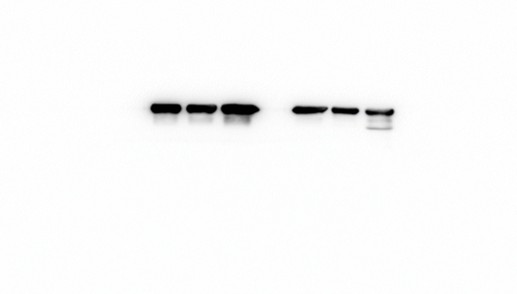


**

**LC3 PHB2**

**PDI FLAG**

**PHB2 β-atin**
